# A Small Secreted Virulence-Related Protein Is Essential for the Necrotrophic Interactions of *Sclerotinia sclerotiorum* with Its Host Plants

**DOI:** 10.1371/journal.ppat.1005435

**Published:** 2016-02-01

**Authors:** Xueliang Lyu, Cuicui Shen, Yanping Fu, Jiatao Xie, Daohong Jiang, Guoqing Li, Jiasen Cheng

**Affiliations:** 1 State Key Laboratory of Agricultural Microbiology, Huazhong Agricultural University, Wuhan, Hubei Province, China; 2 The Provincial Key Lab of Plant Pathology of Hubei Province, College of Plant Science and Technology, Huazhong Agricultural University, Wuhan, Hubei Province, China; University of California Riverside, UNITED STATES

## Abstract

Small, secreted proteins have been found to play crucial roles in interactions between biotrophic/hemi-biotrophic pathogens and plants. However, little is known about the roles of these proteins produced by broad host-range necrotrophic phytopathogens during infection. Here, we report that a cysteine-rich, small protein SsSSVP1 in the necrotrophic phytopathogen *Sclerotinia sclerotiorum* was experimentally confirmed to be a secreted protein, and the secretion of SsSSVP1 from hyphae was followed by internalization and cell-to-cell movement independent of a pathogen in host cells. SsSSVP1^∆SP^ could induce significant plant cell death and targeted silencing of *SsSSVP1* resulted in a significant reduction in virulence. Through yeast two-hybrid (Y2H), coimmunoprecipitation (co-IP) and bimolecular fluorescence complementation (BiFC) assays, we demonstrated that SsSSVP1^∆SP^ interacted with QCR8, a subunit of the cytochrome b-c_1_ complex of mitochondrial respiratory chain in plants. Double site-directed mutagenesis of two cysteine residues (C^38^ and C^44^) in SsSSVP1^∆SP^ had significant effects on its homo-dimer formation, SsSSVP1^∆SP^-QCR8 interaction and plant cell death induction, indicating that partial cysteine residues surely play crucial roles in maintaining the structure and function of SsSSVP1. Co-localization and BiFC assays showed that SsSSVP1^∆SP^ might hijack QCR8 to cytoplasm before QCR8 targeting into mitochondria, thereby disturbing its subcellular localization in plant cells. Furthermore, virus induced gene silencing (VIGS) of QCR8 in tobacco caused plant abnormal development and cell death, indicating the cell death induced by SsSSVP1^∆SP^ might be caused by the SsSSVP1^∆SP^-QCR8 interaction, which had disturbed the QCR8 subcellular localization and hence disabled its biological functions. These results suggest that SsSSVP1 is a potential effector which may manipulate plant energy metabolism to facilitate the infection of *S*. *sclerotiorum*. Our findings indicate novel roles of small secreted proteins in the interactions between host-non-specific necrotrophic fungi and plants, and highlight the significance to illuminate the pathogenic mechanisms of this type of interaction.

## Author Summary

To resist biotrophic and hemibiotrophic phytopathogens, plants utilize an innate immune system, mediated through nucleotide binding (NB)-leucine rich repeat (LRR) proteins, to respond to effectors, most of which are small secreted proteins. Hypersensitive responses (HRs) resulting from this type of interaction can effectively restrain the expansion of biotrophic or hemibiotrophic phytopathogens in plant tissues. However, it is not effective against typical necrotrophs with remarkably broad host range, such as *S*. *sclerotiorum*, because these necrotrophs have long been thought to just simply kill hosts and complete their life cycles using nutrients derived mostly from dead plant tissues. This type of phytopathogen-plant interaction obviously does not comply with the gene-for-gene or inversed gene-for-gene relationship. The results in present study show that SsSSVP1 of *S*. *sclerotiorum* functions as an effector in pathogen-plant interactions. *SsSSVP1* is dramatically induced during infection, and required for the full virulence of *S*. *sclerotiorum*. SsSSVP1 can be internalized by plant cells after being secreted from fungal cells in the absence of a pathogen during infection. Furthermore, SsSSVP1^∆SP^ interacts with QCR8, a subunit of cytochrome b-c_1_ complex, and disturbs the localization of QCR8 in mitochondria, which may disable its biological function. The nonfunctionalization of QCR8 caused significant plant cell death. Hence, SsSSVP1 acts as an effector to manipulate the host cell physiology to facilitate the colonization of *S*. *sclerotiorum*. Obviously, this is a completely different interaction model from the gene-for-gene or inversed gene-for-gene paradigm. These findings suggest that the pathogenesis of *S*. *sclerotiorum* is more subtle and complex than previously appreciated and highlight the significance to investigate the interaction models between the host-non-specific necrotrophs and their hosts.

## Introduction

*Sclerotinia sclerotiorum* (Lib.) de Bary is an exemplary necrotrophic phytopathogenic fungus with a broad host range. At least 408 species of plants are susceptible to this white mold fungus, most of them are from Dicotyledonae but a few are from Monocotyledonae such as onion and garlic [1]. *S*. *sclerotiorum* is also a cosmopolitan pathogen of many economically important crops, including oilseed rape (*Brassica* spp.), sunflowers, soybeans, peanuts and lentils, and its infection often leads to a significant loss of crop production.

Plant pathogens have been categorized as biotrophic, hemibiotrophic and necrotrophic pathogens based on the lifestyles of these agents, and the pathogenic mechanisms are obviously different among the different types of pathogens. Biotrophic pathogens must manipulate host physiology and derive nutrients from living host cells and tissues, whereas hemibiotrophic pathogens absorb nutrients from living cells during the early biotrophic stage of infection and subsequently kill host cells during the later necrotrophic stage of infection. The nutrient acquisition of necrotrophic pathogens is based on host cell killing [2]. Often, biotrophic and hemibiotrophic fungi secrete effectors that manipulate host cell structure and function to obtain nutrients and suppress plant defenses, thereby facilitating infection [3]. The secretion and transfer of effectors into plant host cells are also essential for the pathogenesis of many biotrophic and hemibiotrophic fungi [4–7]. Plant cell death triggered through hypersensitive responses (HRs) is a major obstacle for the further expansion of biotrophic and hemibiotrophic fungi during the initial stage of infection. However, for necrotrophic fungi, host cell death might be beneficial rather than detrimental for pathogenesis; thus, the canonical necrotrophic fungus *S*. *sclerotiorum* secretes a wide array of cell-wall-degrading enzymes (CWDEs) to facilitate host cell wall degrading and ultimately promote infection [8]. As a non-selective phytotoxin, oxalic acid (OA) produced by *S*. *sclerotiorum* can also contribute to pathogenesis in a number of ways (e.g. acidification, chelation of Ca^2+^, low pH activation of degradative enzymes etc.) that augment fungal colonization of host plants [9]. In addition, OA plays a subtle role in the interaction between *S*. *sclerotiorum* and its hosts. For example, OA can suppress the oxidative burst of the host plant [10] and suppress host defenses by manipulating the host redox environment [11]. It also induces apoptotic cell death [12] and plays a crucial role in the control of the interplay of host cell apoptosis and autophagy during infection [13].

Necrotrophic fungi have long been considered as host killers. Previous studies have shown that host-specific necrotrophic fungal pathogens may utilize plant resistance signaling pathways to subvert PCD and enable pathogen growth [14,15]. To date, many interactions between host-specific necrotrophic fungal pathogen effector molecules and their host targets have been reported, including the victorin of *Cochliobolus victoriae* and TRX-h5 as well as LOV1 of *Arabidopsis thaliana* [16], the PC toxin of *Periconia circinata* and *Pc* locus of sorghum [17], the Ptr ToxA of *Pyrenophora tritici-repentis* and *Tsn1* of wheat [14] as well as the SnTox1-Snn1 [18], SnToxA-Tsn1 [19,20], SnTox2-Snn2 [21], SnTox3-Snn3-B1 [22], SnTox4-Snn4 [23], and SnTox3-Snn3-D1 [24] in *Stagonospora nodorum*-wheat pathosystem. These interactions induce a resistance-like response that confers disease susceptibility in an inverse gene-for-gene manner. However, for host-non-specific fungi with remarkably broad host range such as *S*. *sclerotiorum* and *Botrytis cinerea*, emerging evidence suggests that they have more sophisticated and comprehensive strategies for infecting hosts than previously considered. They can manipulate the antagonistic effects between immune pathways to promote disease development in tomato [25]. Actually, even for these kinds of fungi, there is a transition from a biotrophic to necrotrophic lifestyle and the hemi-biotrophic lifestyle may be more temporally and spatially complex than currently depicted [26]. In addition to CWDEs and OA related pathogenic factors, some potential secreted proteinaceous effectors also play crucial roles in the pathogenesis of host-non-specific necrotrophic fungi. For example, we previously reported that a secreted integrin-like protein SSITL of *S*. *sclerotiorum* promotes virulence and directly or indirectly suppresses host resistance during the early stages of infection [27]. Another small secreted protein, Ss-Caf1, functions as a pathogenicity factor to trigger host cell death during the early stages of *S*. *sclerotiorum* infection [28]. Kabbage *et al*. also identified an effector-like protein in *S*. *sclerotiorum* (SsCm1) [13]. The xylanase Xyn11A can induce necrosis independently of the catalytic activity of this enzyme during *B*. *cinerea* infection [29]. However, until recently, there has been little experimental evidence for the existence of the interactions between proteinaceous effectors and host targets for typical necrotrophic phytopathogens, such as *S*. *sclerotiorum* and *B*. *cinerea*. The molecular mechanisms of the interactions between host-non-specific necrotrophic fungal effectors and their host targets is still poorly understood. The identification and characterization of this type of the necrotrophic interactions are difficult because they obviously do not act in the gene-for-gene manner or follow the inverse gene-for-gene scenario.

A recent study reported that the *S*. *sclerotiorum* genome encodes many predicted secreted proteins that might be involved in the interaction between this fungus and its hosts [30]. Notably, in plant-pathogen interactions, most of effectors are small secreted proteins [31–34] except for some non-proteinaceous toxins and secondary metabolites. However, the biological functions of small secreted proteins from many eukaryotic pathogens remain largely unknown. In the present study, we aim at identifying and characterizing proteinaceous effectors which play crucial roles in the interaction between *S*. *sclerotiorum* and its hosts. Digital gene expression profiles (DGE; Solexa/Illumina) and bioinformatics approaches were combined to screen for proteinaceous effector candidates in *S*. *sclerotiorum*. A cysteine-rich, small, secreted protein SsSSVP1 was experimentally confirmed to interact with a component of plant cytochrome b-c1 complex in mitochondrial respiratory chain, which play a crucial role during *S*. *sclerotiorum-*hosts interaction. Our result demonstrated that the necrotrophic fungus *S*. *sclerotiorum* also secretes proteinaceous effectors that has targets in plants and the interaction between these effectors and their targets may seriously disturb the physiological processes of its hosts.

## Results

### SsSSVP1 is a *Sclerotinia-* and *Botryotinia* *-*specific, cysteine-rich, small, secreted protein

In our previous study, the DGE based on deep sequencing technology was used to illuminate the wide range of transcriptional responses associated with six different developmental stages of a virulent wild-type strain, Ep-1PNA367 [35]. In this study, the DGE data was used to identify the differentially expressed genes encoding putative secreted proteins during the vegetative growth stage on PDA and the infection stage on *A*. *thaliana* leaves. There were 314 genes encoding predicted secreted proteins that were identified to be significantly up-regulated during infection (S1 Table). We focused our study on those genes which encode cysteine-rich small proteins. RNAi technique was used to study the biological functions of *S*. *sclerotiorum* genes because of the multinucleated cells of this fungus. Our results showed that silencing *SS1G_02068* significantly reduced the virulence of *S*. *sclerotiorum* and SS1G_02068 (GenBank accession: XM_001597822) could induce significant plant cell death when constitutively expressed in host cells. Thus, we named this protein ‘‘SsSSVP1”, as this is the first report that a small secreted virulence-related protein in *S*. *s* *clerotiorum* that has a target in plant cells.

**Fig 1:**
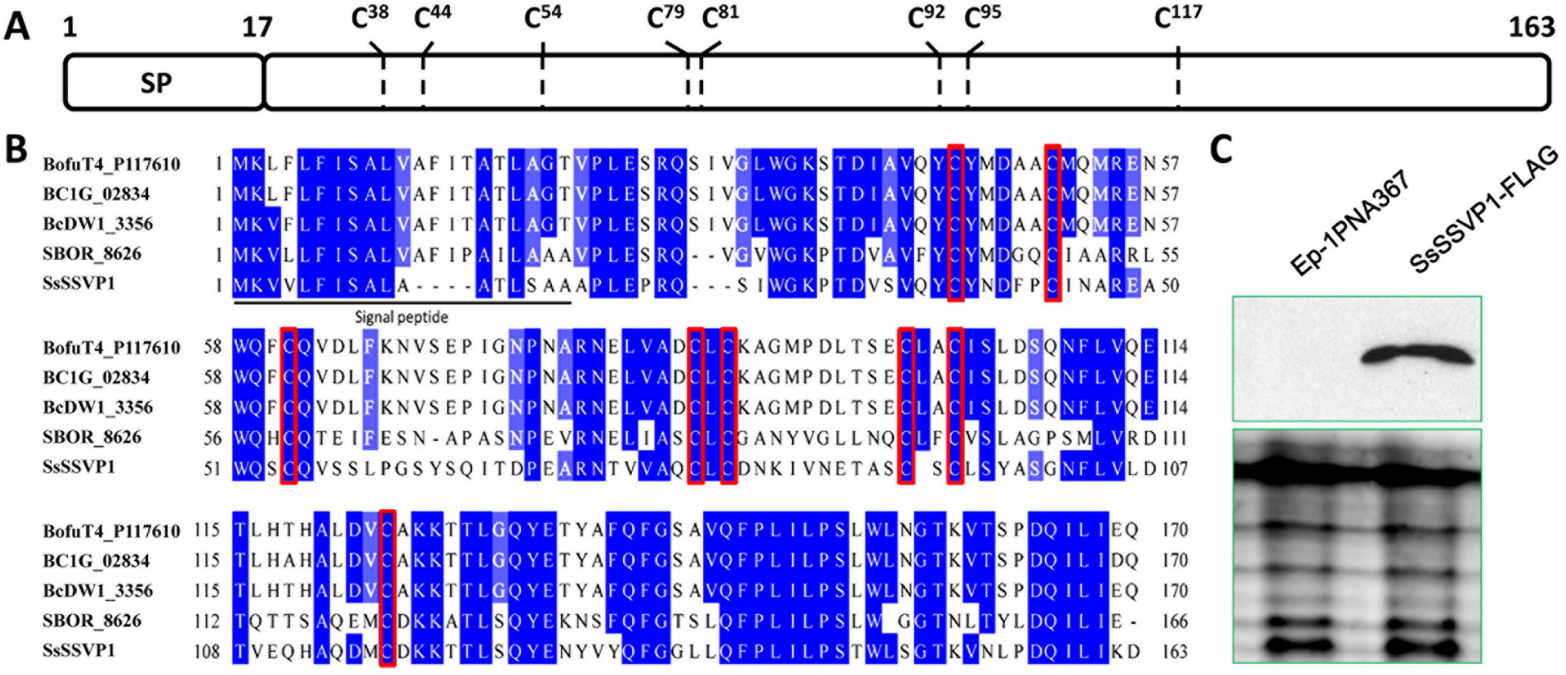
SsSSVP1 is a *Sclerotinia-* and *Botryotinia-*specific, cysteine-rich, small, secreted protein. (**A**) A predicted structure diagram of SsSSVP1 which comprises 163 aa. A putative N-terminal SP (aa 1 to 17) and the position of the eight cysteine residues of SsSSVP1 are present (C^38^, C^44^, C^54^, C^79^, C^81^, C^92^, C^95^ and C^117^). (**B**) Multiple alignments indicate the homologs of SsSSVP1 are only present in *Sclerotinia*- and *Botryotinia* in the organisms sequenced so far and the eight cysteine residues are conserved in these homologs. Red rectangle labels the sites of the eight cysteine residues in multiple alignments. Protein sequences from top to bottom are derived from *B*. *cinerea* T4, *B*. *cinerea* B05.10, *B*. *cinerea* BcDW1, *Sclerotinia borealis* F-4157 and *S*. *sclerotiorum* Ep-1PNA367 respectively. The protein sequences of SsSSVP1 in *S*. *sclerotiorum* Ep-1PNA367 and 1980 are the same. (**C**) Western blot analysis with total proteins isolated from the liquid CM culture of the wild-type strain and SsSSVP1-FLAG engineered strains. SDS-polyacrylamide gel electrophoresis shows the equal loading amount of proteins used for the west blot analysis. Horseradish peroxidase conjugated secondary antibody detected an approximate 17 kDa band in SsSSVP1-FLAG engineered strains, but not in the wild-type strain.

SsSSVP1 is a protein without any known domains which may be specific to *Sclerotinia* and *Botryotinia*, as the homologs of SsSSVP1 have only been identified in *Sclerotinia* and *Botryotinia *in the non-redundant protein sequence database at NCBI to date. SsSSVP1 contains 163 amino acid residues including eight cysteine residues, which account for over 4% (Fig 1A). Multiple sequence alignment indicated that all the cysteine residues in SsSSVP1 are well conserved in its homologues (Fig 1B), indicating these cysteine residues may play an important role in the structure and function of SsSSVP1. Bioinformatics analysis revealed that SsSSVP1 has a predicted N-terminal signal peptide (SP, 1–17 aa), suggesting that it may be a secreted protein (Fig 1A). To test this hypothesis, the FLAG-tagged SsSSVP1 engineered strains were constructed and inoculated in liquid CM medium for shake culture. Western blot result showed that SsSSVP1-FLAG could be detected in the liquid culture medium (Fig 1C), indicating SsSSVP1 is indeed a secreted protein.

### SsSSVP1^∆SP^ can induce significant plant cell death

**Fig 2:**
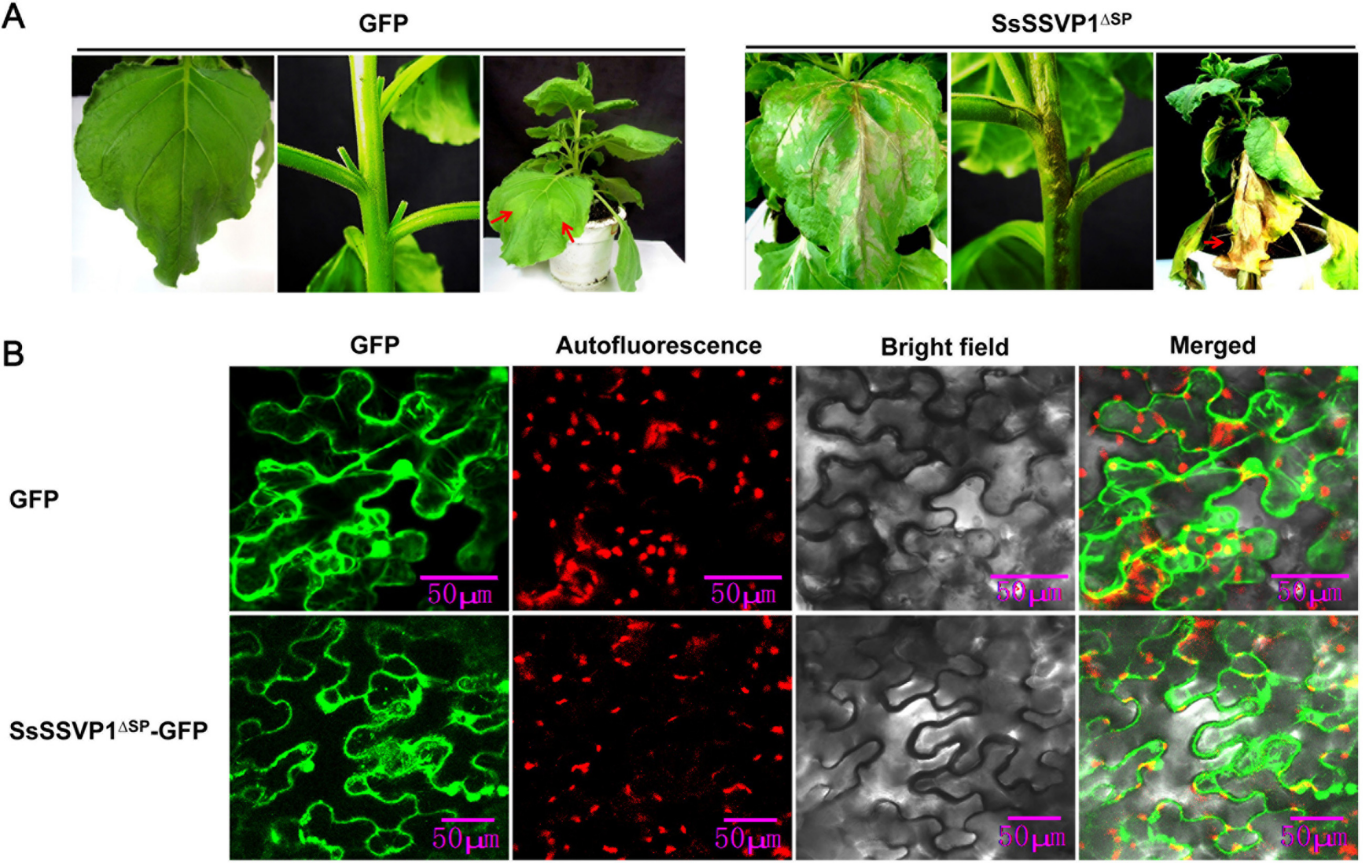
Induction of cell death and the subcellular localization of SsSSVP1^∆SP^ in plant cells. (**A**) SsSSVP1^∆SP^ induces significant systemic plant cell death. *A*. *tumefaciens* containing systemic expression vector pTRV2-SsSSVP1^∆SP^ and pTRV1, respectively, were mixed in equal proportions and infiltrated into lower leaves of the wild-type *N*. *benthamiana*. The leaves and stems were from above the infiltrated sites. Photos were taken 15 days after *A*. *tumefaciens* infiltration. Red arrows indicate infiltration sites. (**B**) Laser confocal micrograph showing SsSSVP1^∆SP^ mainly distributed in the cytoplasm, and especially concentrated in the periphery of cytomembrane. Red particles showed chloroplast autofluorescence. Photos were taken 3 days after agroinfiltration. Maximum projections of 4 confocal images captured along the z-axis are shown.

To characterize the influence of SsSSVP1 over host cells after being secreted, considering a SP is cut off when a secreted protein is secreted from hyphae into plant cells, SsSSVP1^∆SP^ without its SP was constitutively expressed in *Nicotiana benthamiana* using *Agrobacterium tumefaciens*-mediated transformation method. *Agrobacterium* strains carrying the pTRV2-SsSSVP1^∆SP^ virus vector and the pTRV1 vector, the latter of which facilitates the movement of the recombinant virus, were mixed and co-infiltrated into *N*. *benthamiana* leaves. Our result showed that SsSSVP1^∆SP^ could induce significant cell death in leaves, stems and the whole plant (Fig 2A). However, the GFP alone for control did not induce plant cell death, suggesting that plant cell death was specifically induced by SsSSVP1^∆SP^ (Fig 2A). This result indicates SsSSVP1^∆SP^ is toxic to plant cells.

### SsSSVP1 can be internalized into plant cells independently and translocated from cell to cell

**Fig 3:**
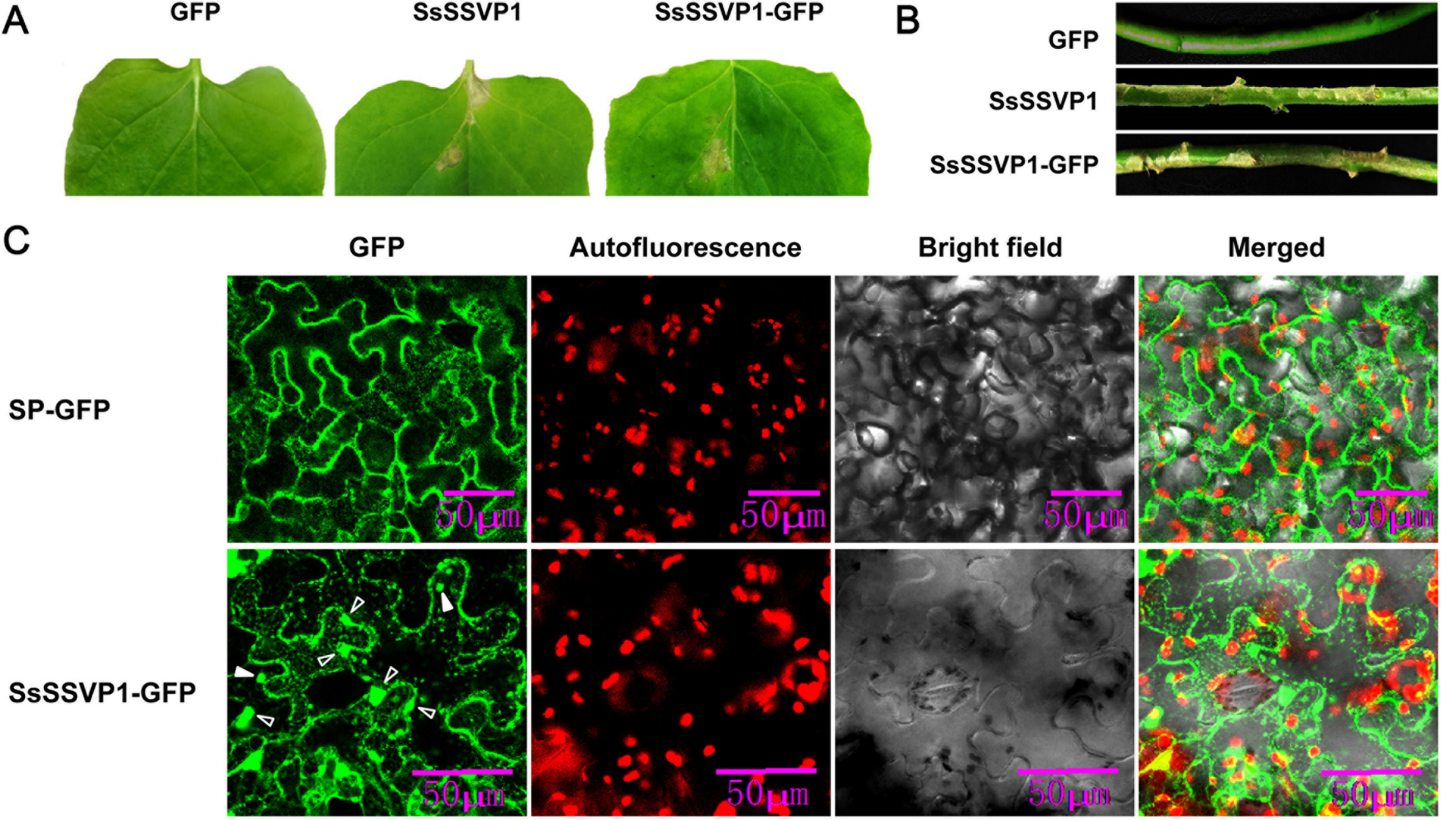
Full SsSSVP1 could still induce plant cell death and it can be internalized into plant cells in the absence of a pathogen. (**A**) SsSSVP1 with SP still can induce cell death in leaves. Upper leaves from above the infiltrated sites were taken photos 10 days after *A*. *tumefaciens* infiltration. (**B**) SsSSVP1 with SP still can induce cell death in stems. GFP alone was used as control. Photos were taken 10 days after *A*. *tumefaciens* infiltration. (**C**) Both SP-GFP (which was used as control) and SsSSVP1 with SP localized in ER-like structure in plant cells (details see S2 Fig), however, only full SsSSVP1 could also localize in cytoplasmic compartments in a particle like form. No particle-like form of SP-GFP was observed in cytoplasm. The SP refers in particular to the SP of SsSSVP1. Red particles show chloroplast autofluorescence. White solid arrows indicate the internalized particle-like form of SsSSVP1-GFP; White hollow arrows show the endocytic vesicle-like structure near plasma membrane. Photos were taken 3 days after agroinfiltration.

A previous report in our lab showed that a small, secreted protein, Ss-Caf1 of *S*. *sclerotiorum* without its SP could induce significant plant cell death, however, full Ss-Caf1 with its SP could not induce plant cell death [28], suggesting that plant cells can recognize SPs from fungi and direct the secretion of fungal proteins expressed in plant cells. Interestingly, we found that full SsSSVP1 with its SP still could induce plant cell death similar to SsSSVP1^∆SP^ (Fig 3A and 3B). So, we postulated that SsSSVP1 could be internalized by plant cells in the absence of a pathogen. If this hypothesis is true, we should still be able to detect SsSSVP1 in plant cells after its secretion. To test this hypothesis, we first examined the subcellular localization of SsSSVP1^∆SP^ in host plant cells. The pTRV2-SsSSVP1^∆SP^-GFP virus vector was constructed and transformed into an *Agrobacterium* strain to conduct infiltration assay on tobacco leaves. Confocal images showed that SsSSVP1^∆SP^ mainly distributed throughout the cytoplasm, particularly concentrated at the periphery of cell membrane (Fig 2B). In addition, SsSSVP1^∆SP^ occasionally localized in nuclei under unknown conditions, and sometimes it scattered in cytoplasm in a particle-like form (S1A Fig). Afterwards, we examined the subcellular localization of SsSSVP1 with its SP and SP-GFP (used for control) in tobacco leaf cells using the same protein expression system. Results showed that both SsSSVP1-GFP and SP-GFP localized in endoplasmic reticulum (ER)-like structure (S2 Fig), however, only SsSSVP1-GFP could be observed to localize in cytoplasmic compartments in a particle-like form, no particle-like form of SP-GFP was observed in cytoplasm, indicating the specificity of the fluorescence signal (Fig 3C). These results indicated the SsSSVP1 could be secreted by plant cells and had plant cell re-entry activity which may result from the internalization of SsSSVP1.

**Fig 4:**
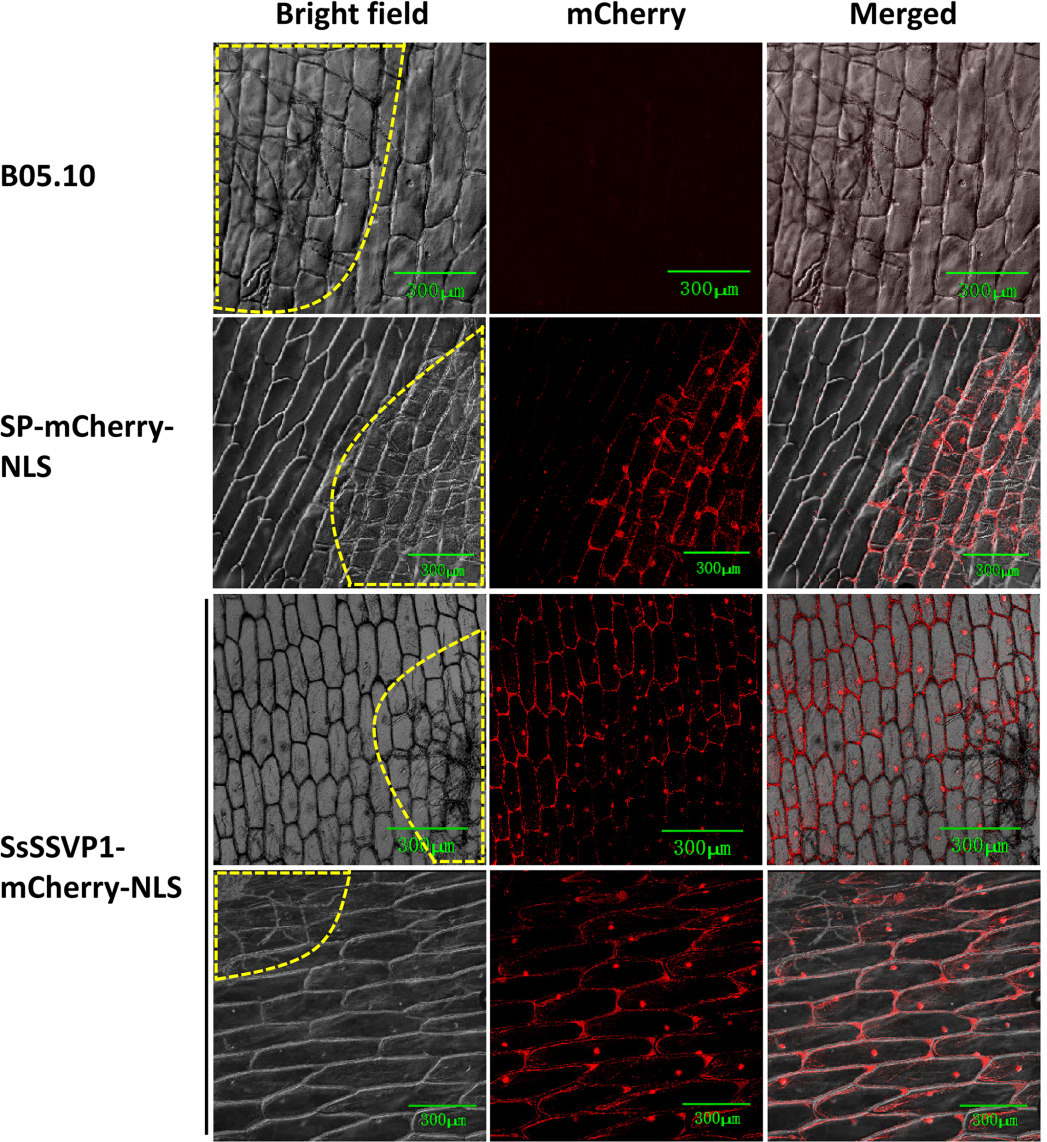
The nuclear targeting-based translocation assay of SsSSVP1-mCherry. *B*. *cinerea* wild-type strain B05.10 and transformants of SP-mCherry-NLS and SsSSVP1-mCherry-NLS constructs were used to perform nuclear targeting assay, and the former two were used as controls. SsSSVP1-mCherry-NLS fluorescence occurred in the nuclei of invaded onion bulb lower epidermal cells and surrounding cells while mCherry-NLS fluorescence occurred only in the nuclei of invaded cells. No fluorescence was observed in the onion tissues infected by B05.10. The same imaging conditions were used in the three channels. Images were taken at 48 hpi using confocal laser scanning microscopy. Different layers of the intact surrounding cells were observed independently to ensure there were no hyphae in these cells. See S3 Fig for an example. The images show maximum projections of 4 confocal images captured along the z-axis. Areas within yellow dotted line indicate hyphal invaded onion epidermal cells.

In order to further confirm that SsSSVP1 can be internalized into plant cells independently, nuclear targeting assay was used to facilitate visualization of the translocation of SsSSVP1 according to Khang et al. [4]. A small nuclear localization signal (NLS) from simian virus large T-antigen [36] was added at the C terminus of the SsSSVP1-mCherry fusion (SsSSVP1-mCherry-NLS) and SP-mCherry fusion (SP-mCherry-NLS, used for control). It is difficult to obtain pure transgenic lines because of the multi-nucleated trait of *S*. *sclerotiorum* and the hyper-virulence of *S*. *sclerotiorum* is not conducive to observe effector translocation, the constructs described above were transformed into *B*. *cinerea* (which is phylogenetically close to *S*. *sclerotiorum*) to more easily visualize faint fluorescence. The result showed that the SP-mCherry-NLS fluorescence was only observed in the nuclei of infected host cells but not the neighboring host cells, however, the SsSSVP1-mCherry-NLS fluorescence was observed in the nuclei of infected host cells and intact surrounding host cells (Fig 4). All these intact surrounding host cells were checked in different layers using z-axis scanning of a confocal laser microscope to ensure there were no hyphae in these cells (S3 Fig). These results further indicated SsSSVP1 can be internalized into plant cells independently and move from cell to cell like effectors in other hemibiotrophic fungi [4].

### *SsSSVP1* plays a crucial role in virulence

**Fig 5:**
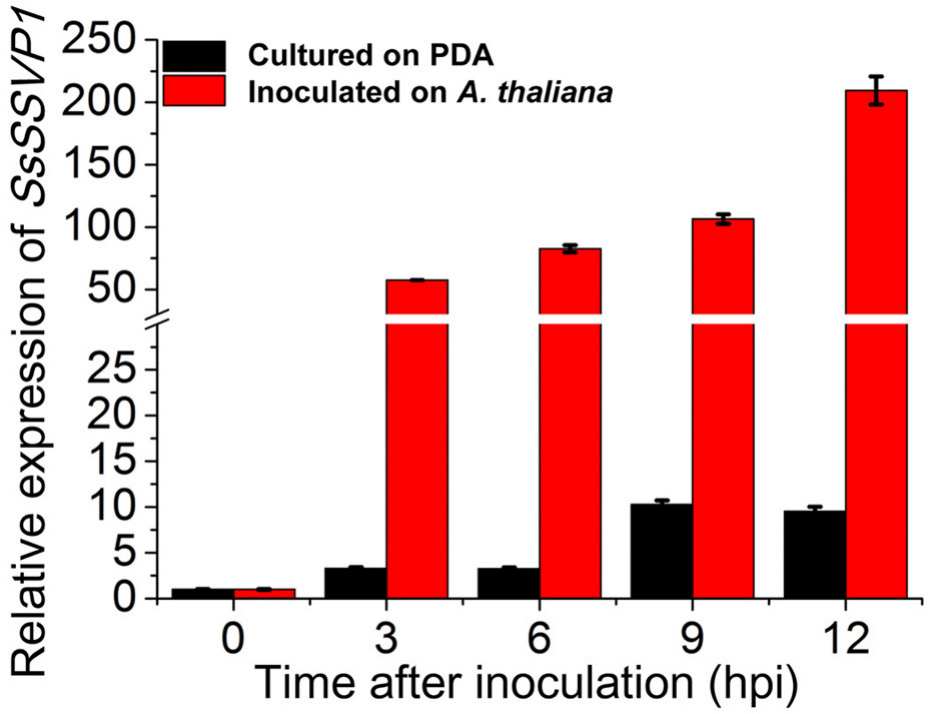
Gene expression analysis of *SsSSVP1* in the wild-type strain Ep-1PNA367 during infection. The relative expression of *SsSSVP1* is significantly up-regulated during the early stages of infection in *A*. *thaliana* (Col-0) leaves (red columns) compared to that during vegetative growth on PDA (dark columns). The expression level at 0 hpi on PDA was set as 1.0. The expression levels of β-tubulin are used to normalize the expression levels of *SsSSVP1* in different samples. Three independent replicates were performed. The bars represent the mean relative expression of *SsSSVP1* ± the standard deviation of the mean.

**Fig 6:**
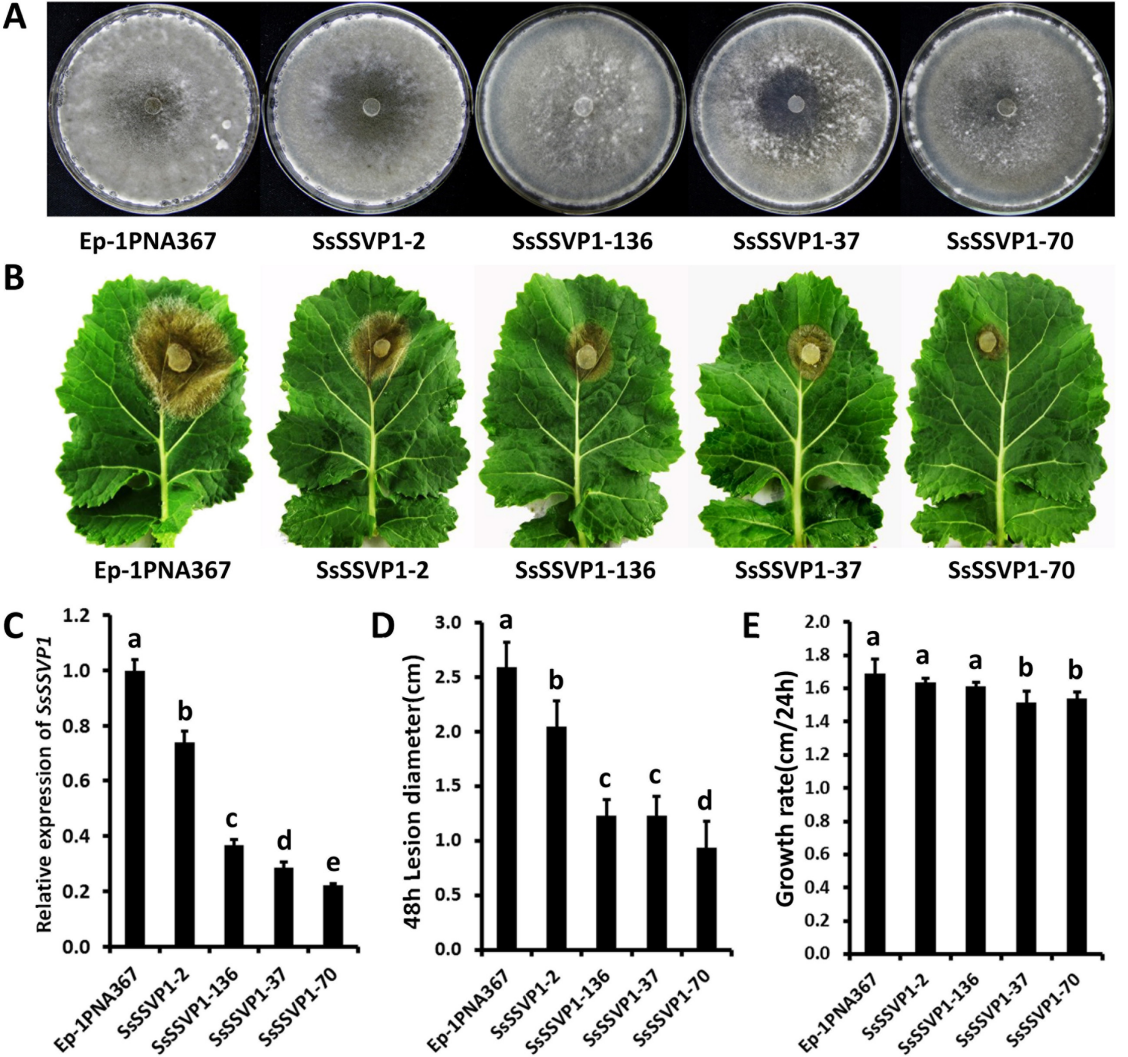
Phenotypes of *SsSSVP1*-silenced transformants of *S*. *sclerotiorum*. (**A**) The colony morphology of *SsSSVP1-*silenced transformants. Colonies were grown on PDA for 10 days at 20°C. (**B**) *SsSSVP1*-silenced transformants showed significantly reduced virulence on detached oilseed rape (*B*. *napus* zhongyou 821) leaves. Virulence was evaluated on detached oilseed rape leaves according to the lesion diameter. Photos were taken at 48 hpi. (**C**) The relative expression of *SsSSVP1* in silenced transformants was determined through qRT-PCR. The expression levels of β-tubulin were used to normalize the expression levels of *SsSSVP1* in the different samples. The expression level in the wild-type strain was set as 1.0. (**D**) Comparison of the lesion diameter of silenced transformants and the wild-type strain. (**E**) Comparison of the growth rate of silenced transformants and the wild-type strain. In all experiments, three independent replicates were performed. The values are presented as the means±s.d. Different letters on the same graph indicate statistical significance, P = 0.05.

Quantitative reverse transcription PCR (qRT-PCR) analysis showed that when pure actively growing hyphal fragments of *S*. *sclerotiorum* without culture medium were inoculated onto the leaves of *A*. *thaliana* (Col-0), the transcript levels of *SsSSVP1* rapidly increased by more than 50-fold at 3 hours post inoculation (hpi) and then gradually increased during the later infection stages (6–12 hpi, Fig 5). This result is consistent with the DGE data and suggests that *SsSSVP1* may be involved in infection of *S*. *sclerotiorum*. In order to explore the roles of SsSSVP1 in virulence of *S*. *sclerotiorum*, RNAi technology was used because of the multi-nucleated cells. QRT-PCR was used to examine the transcript accumulation in *SsSSVP1-*silenced transformants. Three transformants (SsSSVP1-136, SsSSVP1-37 and SsSSVP1-70) showing dramatically reduced *SsSSVP1* expression and one transformant (SsSSVP1-2) with a slightly reduced *SsSSVP1* expression (Fig 6C) were selected for further study. The colony morphology, virulence and growth rate of these transformants were compared to the wild-type strain Ep-1PNA367 (Fig 6A, 6B, 6D and 6E). The virulence of *SsSSVP1*-silenced mutants was significantly reduced, and only small lesions were developed on the detached *Brassica napus* leaves at 2 days post inoculation (dpi). For example, on average of three independent experiments, lesions induced by SsSSVP1-70 were approximately 0.9 cm in diameter, while lesions induced by the wild-type strain were approximately 2.6 cm in diameter. Furthermore, the decreases in virulence were positively correlated with the silencing efficiency (Fig 6C and 6D), indicating the virulence reduction of the silenced transformants was caused by the silencing of *SsSSVP1*. *In vivo* inoculation assay showed the virulence of *SsSSVP1*-silenced mutants was also dramatically reduced on *A*. *thaliana* leaves compared to that of the wild-type strain (S4A and S4B Fig), indicating the virulence reduction of *SsSSVP1*-silenced mutants is not host-specific. Although the growth rate of *SsSSVP1*-silenced transformants was slightly reduced compared to that of wild-type strain (Fig 6E), statistical analysis indicated the expression reduction of *SsSSVP1* had more effect on virulence than growth rate, which means the virulence reduction is not intimately associated with growth rate.

In order to further investigate the biological functions of SsSSVP1, *S*. *sclerotiorum* transformants over-expressing SsSSVP1-FLAG were used to perform virulence assay. QRT-PCR results showed that increase in the expression of *SsSSVP1* varied in different transformants (S5C Fig). Western blot analysis showed that the SsSSVP1-FLAG could be detected in total protein extracts from the mycelia of these over-expression transformants, of which the OESsSSVP1-3 was used as an example (S5D Fig). However, there was no obvious difference between colonial morphology, virulence and growth rate of the over-expression transformants and the wild-type strain (S5A, S5B, S5E and S5F Fig). The rapid increase of *SsSSVP1* expression level in the wild-type strain during infection could possibly explain the lack of difference in virulence between *SsSSVP1*-overexpression strains and the wild-type strain.

### SsSSVP1^∆SP^ forms homo-dimer and interacts with QCR8

**Fig 7:**
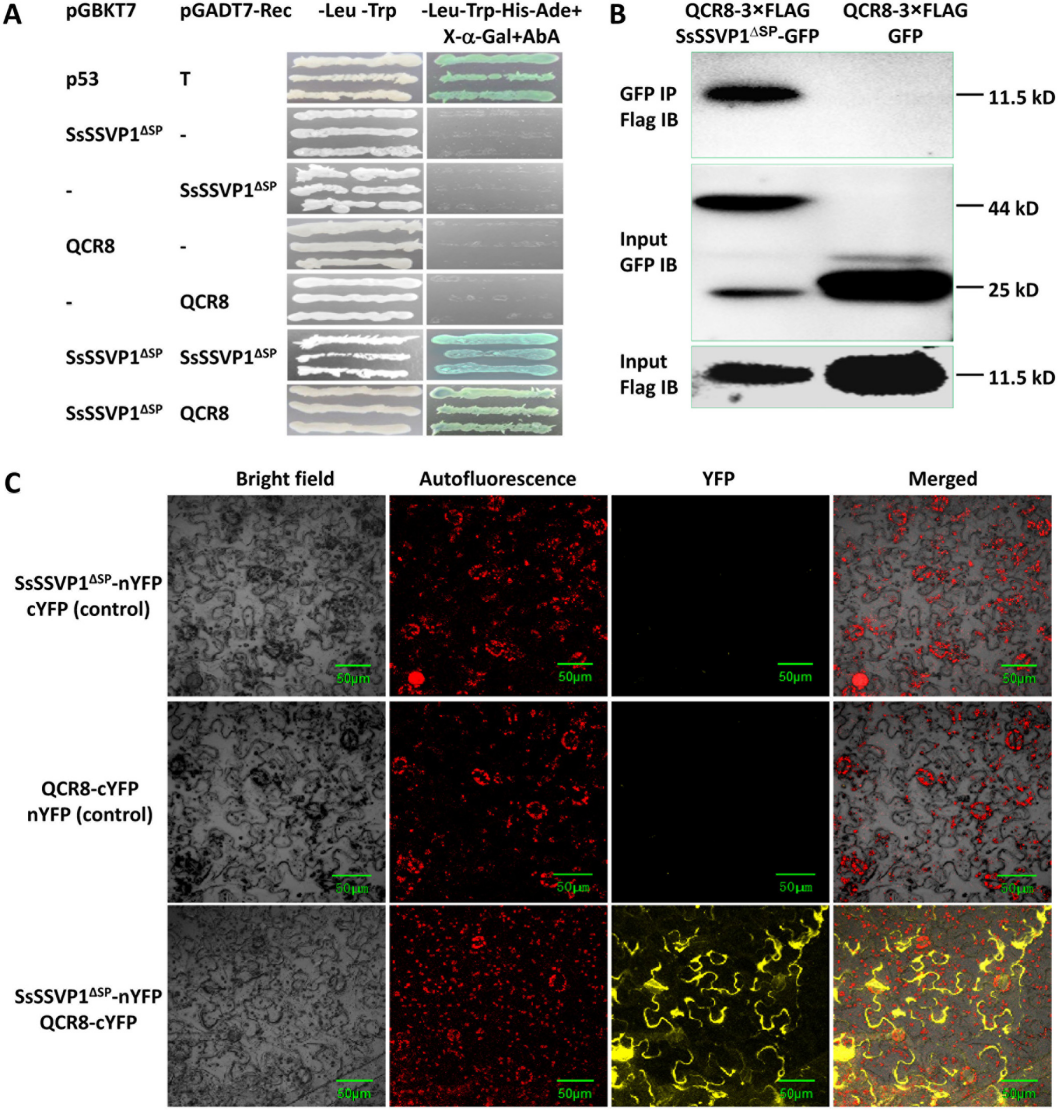
SsSSVP1^∆SP^ can form homo-dimer and interact with QCR8 of *A*. *thaliana*. (**A**) Y2H assay showed SsSSVP1^∆SP^ formed a homo-dimer and interacted with QCR8. pGBKT7-53 and pGADT7-T (Clontech) were used as positive control for protein-protein interaction. “-” means there is an empty vector. The negative controls indicated SsSSVP1^∆SP^ and QCR8 were not self-activated. Photos were taken 2 dpi. (**B**) Co-IP assay for SsSSVP1^∆SP^ and QCR8 interaction in *N*. *benthamiana* leaves. GFP-tagged SsSSVP1^∆SP^ but not GFP alone interacts with 3×FLAG-tagged QCR8. IP = immunoprecipitation, IB = immunoblot. (**C**) BiFC confirms SsSSVP1^∆SP^ interacts with QCR8 in cytoplasm of plant cells. Red particles show chloroplast autofluorescence. Fluorescence was monitored 3 days after agroinfiltration using confocal laser scanning microscopy. The images show maximum projections of 4 confocal images captured along the z-axis.

To further understand how *SsSSVP1* affects the virulence of *S*. *sclerotiorum*, yeast two-hybrid (Y2H) technique was used to screen an *A*. *thaliana* cDNA library to identify the targets that interact with SsSSVP1^∆SP^ in plants. Our Y2H assay showed that SsSSVP1^∆SP^ interacted with itself (Fig 7A), indicating SsSSVP1^∆SP^ may function in plant cells in the form of homo-dimer. Meanwhile, our results demonstrated that SsSSVP1^∆SP^ could interact with QCR8 (AT3G10860), the subunit 8 of cytochrome b-c_1_ complex which is the component of mitochondrial respiratory chain (Fig 7A). The *QCR8* gene is well conserved in plants, and our Y2H assay further showed that SsSSVP1^∆SP^ could interact with all the homologs of QCR8 in *A*. *thaliana* and *N*. *benthamiana* (Fig 7A and S6 Fig), indicating the possible universal existence of this necrotrophic interaction during the infection of *S*. *sclerotiorum* on many hosts. To determine if SsSSVP1^∆SP^ interacts with QCR8 in plant tissues, we co-expressed the GFP-tagged SsSSVP1^∆SP^ and 3×FLAG-tagged QCR8 in *N*. *benthamiana* leaves by *A*. *tumefaciens* infiltration method, our co-immunoprecipitation (co-IP) assay also supported that SsSSVP1^∆SP^ interacted with QCR8 (Fig 7B). Furthermore, this result was further confirmed *in planta* using the bimolecular fluorescence complementation (BiFC) technique. SsSSVP1^∆SP^-nYFP (N-terminal yellow fluorescent protein fragment) and QCR8-cYFP (C-terminal yellow fluorescent protein fragment) were transiently co-expressed in *N*. *benthamiana* leaves. Yellow fluorescence was detected in cytoplasm, especially at the periphery of cell membrane (Fig 7C), suggesting that SsSSVP1^∆SP^ interacts with QCR8 in plant cell cytoplasm.

### C^38^ and C^44^ are essential for function of SsSSVP1^∆SP^

**Fig 8:**
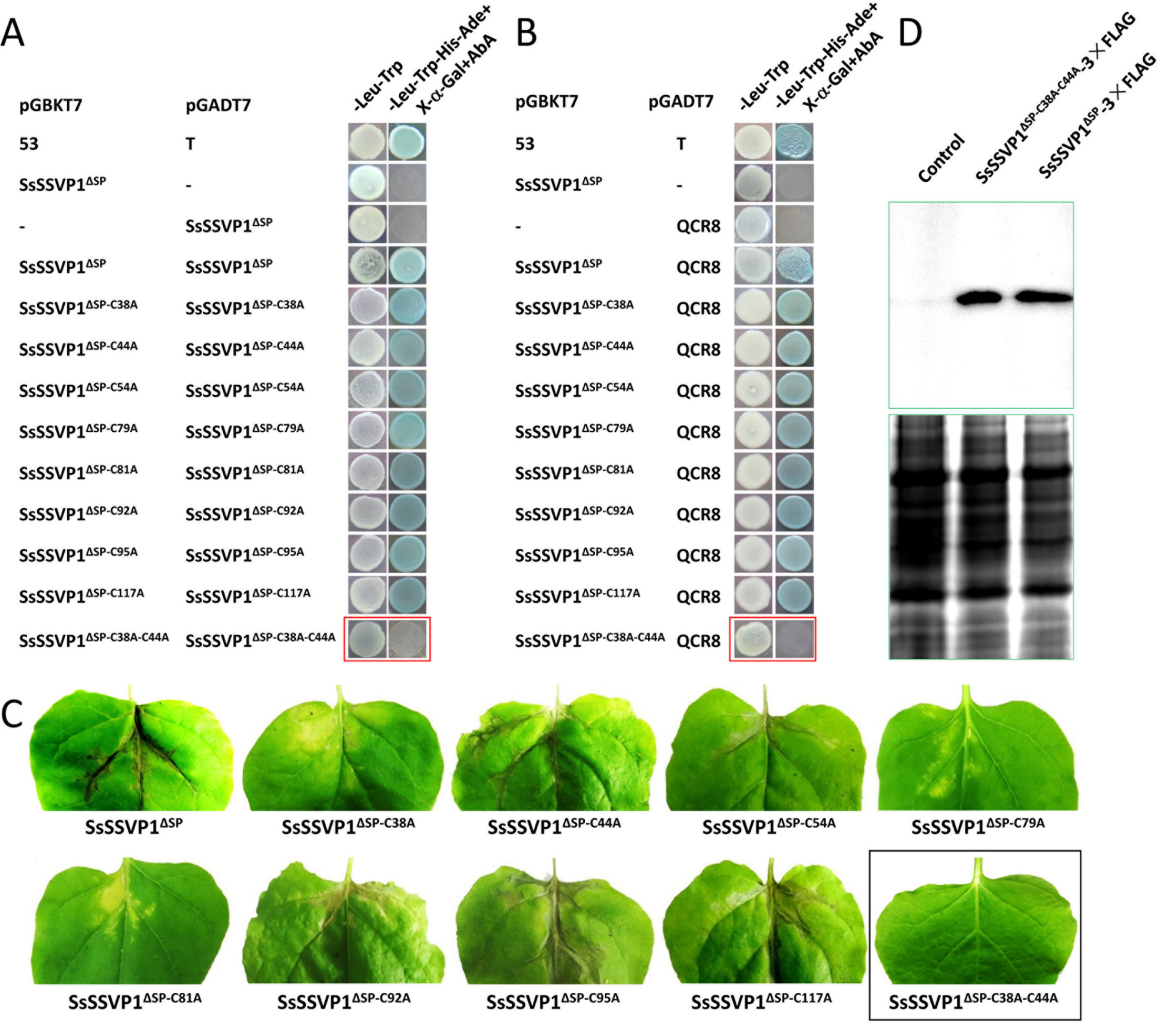
C^38^ and C^44^ play crucial roles in the dimer formation, the SsSSVP1^∆SP^-QCR8 interaction and the biological function of SsSSVP1^∆SP^. The functional analysis of the eight cysteine residues of SsSSVP1^∆SP^ in its dimer formation and interaction with QCR8. Y2H result showed single site-directed mutation of the eight cysteine residues had little effect on the dimer formation (**A**) and the interaction of SsSSVP1^∆SP^ with QCR8 (**B**). However, if C^38^ and C^44^ were simultaneously substituted with alanine, the double-point mutant SsSSVP1^∆SP-C38A-C44A^ could not form dimer (**A**) and interact with QCR8 (**B**), indicated in red rectangles. Photos were taken 2 dpi. pGBKT7-53 and pGADT7-T were used as positive controls (Clontech). (**C**) Single site-directed mutation of the eight cysteine residues of SsSSVP1^∆SP^ had little effect on induction of plant cell death but the double-point mutant SsSSVP1^∆SP-C38A-C44A^ lost the capability to induce plant cell death, indicated in black rectangle. The single- and double-point mutants of SsSSVP1^∆SP^ were expressed in tobacco leaves individually through *A*. *tumefaciens-*mediated plant transformation method. Photos were taken 10 days after *A*. *tumefaciens* infiltration. (D) Western blot analysis showed that horseradish peroxidase conjugated secondary antibody could detect an approximate 19 kDa band in tobacco leaf cells expressing SsSsSSVP1^∆SP^-3×FLAG and SsSsSSVP1^∆SP-C38A-C44A^-3×FLAG but not in control (tobacco leaf cells infiltrated with *A*. *tumefaciens* with empty vector). SDS-polyacrylamide gel electrophoresis shows the equal loading amount of proteins used for the west blot analysis.

As described above, many effectors are cysteine-rich proteins. Additionally, the eight cysteine residues are well conserved in the homologs of SsSSVP1. In order to examine if these cysteine residues play crucial roles in the function of SsSSVP1, single site-directed mutagenesis of the eight cysteine residues was conducted in SsSSVP1^∆SP^. Our results showed that all the single-point mutations had little effects on the dimer formation of SsSSVP1^∆SP^ (Fig 8A), and the interaction between SsSSVP1^∆SP^ and QCR8 (Fig 8B). In addition, the expression of SsSSVP1^∆SP^ with all single-point mutations still could induce plant cell death (Fig 8C). However, our Y2H and single site-directed mutagenesis combined assays showed that SsSSVP1^∆SP-C38A^ could not interact with SsSSVP1^∆SP-C44A^ (S7 Fig). Furthermore, double-point mutation at residues 38 (C to A) and 44 (C to A) made SsSSVP1^∆SP^ lose the ability to interact with itself and with QCR8 (Fig 8A and 8B). These results further indicated the specificity of homo-dimer formation of SsSSVP1^∆SP^ and the interaction between SsSSVP1^∆SP^ and QCR8. Meanwhile, SsSSVP1^∆SP-C38A-C44A^ also could not induce plant cell death any more (Fig 8C), although it could express well and was stability in plant (Fig 8D). These results indicated C38 and C44 play a crucial role in maintaining the structure and biological functions of SsSSVP1.

### Interaction between SsSSVP1^∆SP^ and QCR8 disturbs the subcellular localization of QCR8 in mitochondria

**Fig 9:**
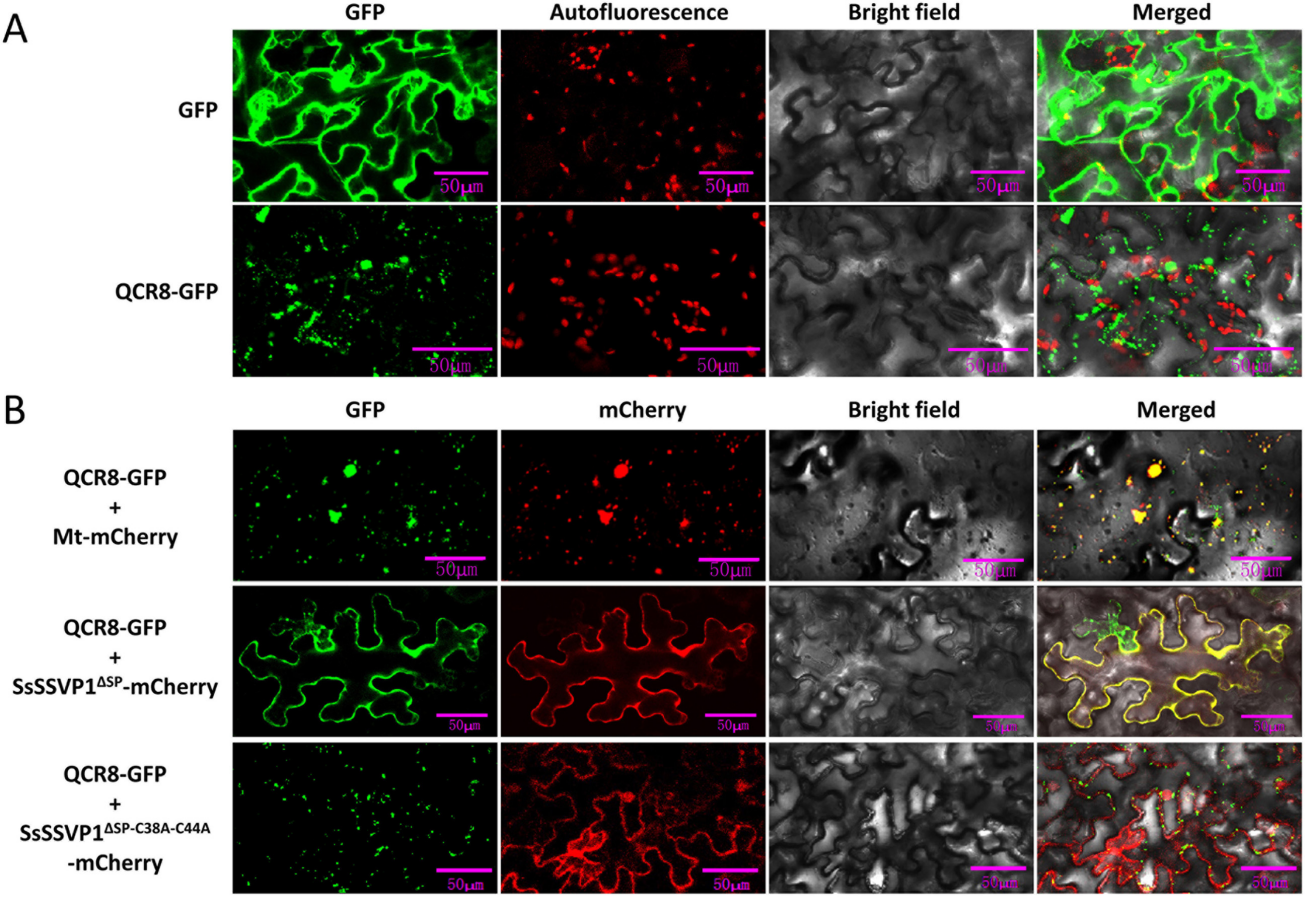
The interaction between SsSSVP1^∆SP^ and QCR8 disturbs the subcellular localization of QCR8 in mitochondria. (**A**) The fluorescent localization of QCR8 in mitochondria. The red particles show chloroplast autofluorescence. (**B**) SsSSVP1^**∆SP**^ hijacks QCR8 into cytoplasm before it targets to mitochondria. QCR8 and mitochondria marker (Mt-mCherry) co-localize in mitochondria, while QCR8 and SsSSVP1^∆SP^ most commonly co-localize in cytoplasm. However, the double site-directed mutant SsSSVP1^∆SP-C38A-C44A^ losing the ability to interact with QCR8 did not affect the subcellular localization of QCR8. Fluorescence was monitored 3 days after agroinfiltration using confocal laser scanning microscopy. The images show maximum projections of 4 confocal images captured along the z-axis.

Our BiFC result showed that SsSSVP1^∆SP^ interacted with QCR8 in cytoplasm, especially at the periphery of cell membrane. However, QCR8 is one subunit of cytochrome b-c_1_ complex, which localizes in mitochondria [37], so we hypothesize the interaction between SsSSVP1^∆SP^ and QCR8 might change the native subcellular localization of QCR8, and SsSSVP1^∆SP^ could hijack QCR8 to cytoplasm. To test this hypothesis, SsSSVP1^∆SP^-mCherry and QCR8-GFP were co-expressed in *N*. *benthamiana* leaves using *Agrobacterium* infiltration method for the observation of their co-localization. As expected, QCR8 alone localized in mitochondria (Fig 9A), because it co-localized with the mitochondria-mcherry marker [38] (Fig 9B). However, SsSSVP1^∆SP^ and QCR8 co-localized in cytoplasm (Fig 9B), which is in accordance with the BiFC results. Additionally, QCR8 still localized in mitochondria when it was co-expressed with the double site-directed mutant SsSSVP1^∆SP-C38A-C44A^ losing the ability to interact with QCR8 (Fig 9B), indicating the specificity of fluorescence distribution of the SsSSVP1^∆SP^ and QCR8 co-localization. Occasionally, the co-localization of SsSSVP1^∆SP^ and QCR8 in nuclei or in cytoplasmic compartments of plant cells could also be observed (S1B Fig). QCR8 is encoded by nuclear genome and translated in cytoplasm. Our results indicated that the interaction between SsSSVP1^∆SP^ and QCR8 could disturb the native localization of QCR8, and SsSSVP1^∆SP^ might hijack QCR8 to cytoplasm before QCR8 was translocated into mitochondria.

### Silencing of *QCR8* led to plant abnormal development and cell death

**Fig 10:**
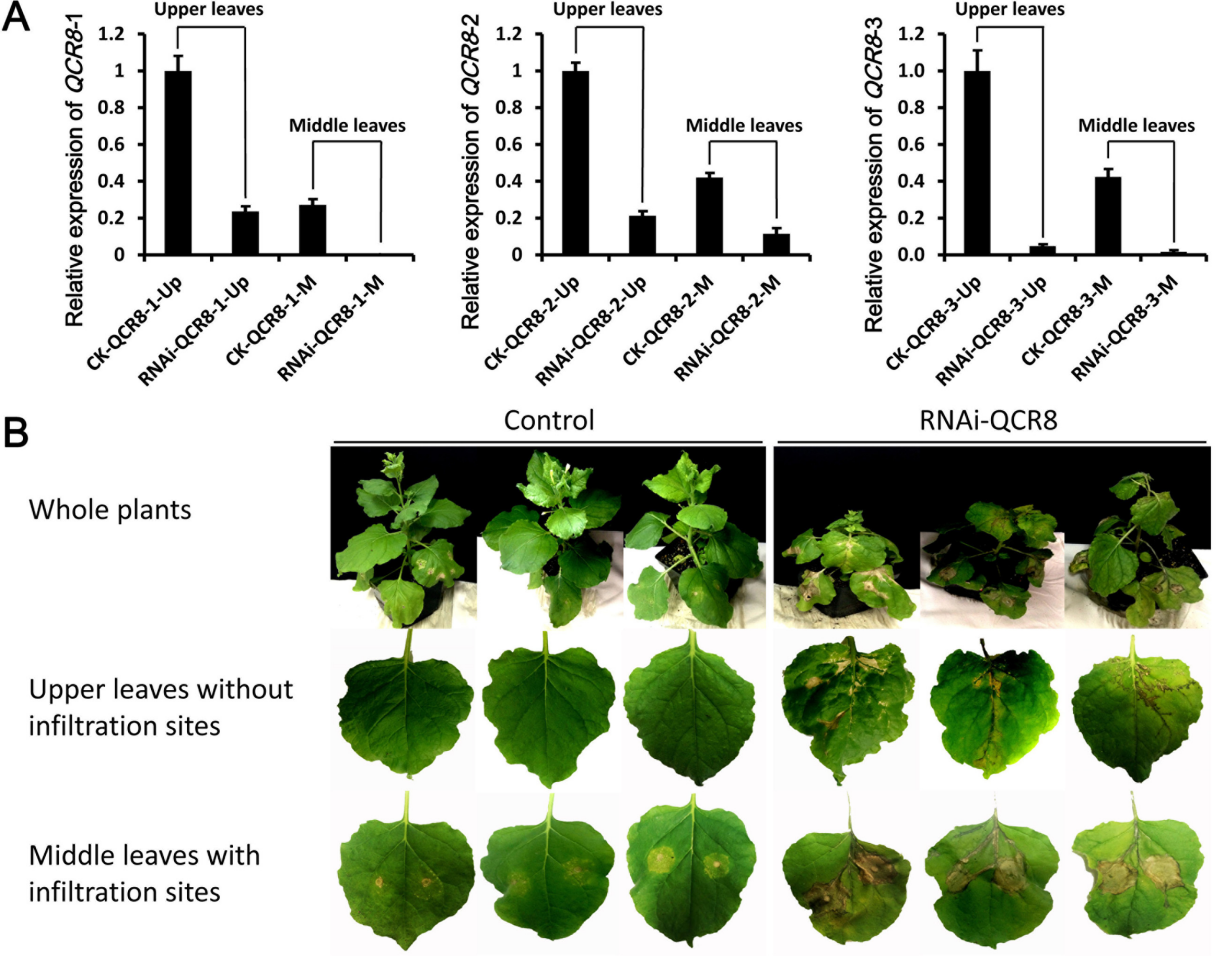
Silencing of *QCR8* leads to plant abnormal development and cell death. (**A**) The relative expression levels of three *QCR8* genes (*QCR8-1*, *QCR8-2* and *QCR8-3*) in silenced *N*. *benthamiana* lines were determined through qRT-PCR. The expression levels of the actin gene in *N*. *benthamiana* were used to normalize the expression levels of *QCR8*. The *QCR8* expression level in the control lines was set as 1.0. This qRT-PCR assay was performed one month after *A*. *tumefaciens* infiltration. “Up” and “M” indicated these samples were from the upper leaves and middle leaves, respectively. “CK” and “RNAi” indicated these samples were from the control lines and the silenced lines which were infiltrated with *A*. *tumefaciens* containing empty vectors and silencing vectors, respectively. (**B**) The phenotype of *QCR8*-silenced *N*. *benthamiana* lines (RNAi-QCR8) using TRV based VIGS system. The lines transformed using *A*. *tumefaciens* containing VIGS-pTRV2 empty vector were used as control. Silencing of the *QCR8* in *N*. *benthamiana* plants caused growth retardation and cell death. No obvious phenotype was observed in the control. Photos were taken 10 days post *A*. *tumefaciens* infiltration.

Our co-localization and BiFC assays showed the SsSSVP1^∆SP^-QCR8 interaction disturbed the subcellular localization of QCR8, which might disable the biological functions of QCR8. To test this hypothesis, a tobacco rattle virus (TRV)-based virus induced gene silencing (VIGS) system [39] was used to knock-down the three homologs of QCR8 encoding genes in *N*. *benthamiana*. The endogenous tobacco phytoene desaturase gene (*PDS*) was used to examine the effectiveness of the TRV-VIGS system (S8 Fig). QRT-PCR results showed that the transcript abundance of the three QCR8 encoding genes was reduced in both upper leaves and middle leaves of the silenced lines in varying degrees, compared to that in control lines (Fig 10A). Targeted silencing of QCR8 resulted in stunted development of stem apex which caused most of the QCR8-silenced plants to exhibit dwarf phenotype (Fig 10B). More importantly, approximately 78.9% (45/57) of the silenced lines showed plant cell death phenotype on the leaves with and without infiltration sites. No control lines exhibited these phenotypes. Together with the data that the double site-directed mutant SsSSVP1^∆SP-C38A-C44A^ cannot interact with QCR8 and also lost the capability to induce plant cell death, these results indicated the plant cell death induced by SsSSVP1^∆SP^ might be caused by the SsSSVP1^∆SP^-QCR8 interaction, which disturbed the subcellular localization of QCR8 and hence made the QCR8 lose its biological function.

## Discussion

*S*. *sclerotiorum* is a typical necrotrophic fungal pathogen that produces oxalic acid and CWDEs to kill plant cells and subsequently feeds on the dead tissues. However, increasing evidence suggests the pathogenesis of *S*. *sclerotiorum* is more complex than originally considered. In this study, a *Sclerotinia-* and *Botryotinia-*specific, small, secreted protein SsSSVP1 was identified, and its biological functions in the interactions between *S*. *sclerotiorum* and its hosts were explored. SsSSVP1 is a cysteine-rich protein which is predicted to form disulfide bonds intramolecularly. The cysteine residues are essential for the formation of disulfide bonds, which may facilitate the formation of stable homodimers, heterodimers, homopolymers, or heteropolymers, suggesting the important roles for these residues in protein folding and in maintaining the structural stability of some secreted proteins [40,41], particularly those are secreted into the oxidizing environment of extracellular medium [40]. The single site-directed mutagenesis of the eight cysteine residues in SsSSVP1^∆SP^ had little effects on its structure and function because the mutants still can form homo-dimer, interact with QCR8 and induce plant cell death. Although it seemed that the degree of cell death induced by different single-point mutants of SsSSVP1^∆SP^ varied at the early stage (10 dpi) after *A*. *tumefaciens* infiltration, the plant cells eventually died at the late stage (30 dpi). The difference of the degree of cell death at the early stage may be due to different plant growth status and different transmission speed of the virus. However, the double-point mutant SsSSVP1^∆SP-C38A-C44A^ could not induce plant cell death no matter at the early stage or at the late stage after *A*. *tumefaciens* infiltration. Meanwhile, SsSSVP1^∆SP-C38A-C44A^ also could not form homo-dimer or interact with QCR8. The dimer formation may be very important for SsSSVP1 when it is exposed to plant intercellular space during infection. This molecular mechanism has significant meaning in many cysteine-rich proteinase inhibitors, where even cleavage of the reactive site peptide bond does not change its overall conformation and such “modified” inhibitor still possesses antiproteinase activity [42]. QCR8 does not have any cysteine residues, indicating the interaction between SsSSVP1^∆SP^ and QCR8 is not maintained by intermolecular disulfide bonds but by their respective tertiary structure. In conclusion, these results indicated there might be at least two disulfide bonds maintaining the tertiary structure of SsSSVP1 intramolecularly, affecting the stability and rigidity of this small secreted protein. Our results suggested C^38^ and C^44^ were essential to maintain the structure and function of SsSSVP1^∆SP^, however, we do not rule out the case that the other cysteine residues also play important roles.

A primary role of effectors is to inhibit host defense mechanisms [43–45]. However, the roles of effectors in biotrophic and necrotrophic fungi might be different, as the former require live host tissues, while the latter prefer dead plant tissues. Most effectors in biotrophic fungi suppress programmed cell death [46] while many effectors in necrotrophic fungi induce plant cell death. Our results also indicated that SsSSVP1^∆SP^ induced significant plant cell death. In different repeated tests, SsSSVP1^∆SP^-GFP always mainly localized in the plant cytoplasm, occasionally localized in cytoplasmic compartments in a particle-like form or in nuclei in different areas even in the same infiltrated tobacco leaf. The difference in the fluorescence distribution in the plant cells expressing SsSSVP1^∆SP^ might be due to the fact that the cells are at different stages of apoptosis. The mechanisms of the translocation of RXLR effectors in oomycetes or RXLR-like variants in fungi into plant cells has been documented and discussed [47–57], however, the mechanisms underlying the delivery in host cells of fungal effectors without RXLR motif are poorly understood, although the phenomena of the internalization and cell-to-cell movement of some fungal effectors were observed previously [4,58]. Previous research showed Ptr ToxA produced by *P*. *tritici-repentis* may be internalized via receptor-mediated endocytosis (RME) by sensitive wheat mesophyll cells and the endocytic vesicle-like structure was observed near plasma membrane [58]. Ptr ToxA is compartmentalized after internalization and forms particle-like structures in plant cells [58]. Interestingly, similar situations were observed in SsSSVP1 (Fig 3C). Hence, we infer that SsSSVP1 and Ptr ToxA have similar cell entry mechanism. In the case of Ptr ToxA, one motif Arg-Gly-Asp (RGD) was predicted to be involved in its interaction with a putative integrin-like receptor in the host [59,60]. However, neither an RGD-like motif nor an RXLR-like motif was found in SsSSVP1. The exact molecular mechanism of SsSSVP1 crossing the plant plasma membrane from the apoplastic space to the interior of plant cells in the absence of a pathogen should be explored in future. Additionally, the cell-to-cell movement of SsSSVP1 is likely the result of the internalization and translocation of SsSSVP1 in the host apoplastic space, because the fluorescent signal could be clearly detected in the apoplastic space of the surrounding cells of the invaded host cells (Fig 4).

QCR8 is a subunit of the cytochrome b-c_1_ complex comprising 10 different polypeptide subunits in plants [61]. The cytochrome b-c_1_ complex is the center component of the mitochondrial respiratory chain, coupling the transfer of electrons from ubihydroquinone to cytochrome c with the generation of a proton gradient across the mitochondrial membrane [37]. We found that SsSSVP1^∆SP^ could hijack QCR8 into the cytoplasm of plant cells and disturb the native localization of QCR8 in mitochondria. This character of SsSSVP1 is similar to that of a rice stripe virus (RSV) specific protein RSV SP, which hijacks host PsbP into cytoplasm from chloroplast [62]. Although we do not know that if the deletion of QCR8 is lethal to plants, our results showed silencing of QCR8 caused obvious plant cell death. This phenomenon indicated a link between the SsSSVP1^∆SP^-QCR8 interaction and the biological function loss of QCR8. Alteration of QCR8 native subcellular localization or lack of QCR8 may eventually affect the energy metabolism of plant cells, because knock-down of *QCR8* significantly affected plant growth and development. Obviously, the interaction model of SsSSVP1^∆SP^ and QCR8 is very different from that of classic effectors and *R* genes. The ‘gene for gene’ and reverse ‘gene for gene’ model might not apply to this typical necrotrophic fungi-host interaction system, as there are almost no resistant hosts to these canonical necrotrophic pathogens. On the other hand, the components of the cytochrome b-c_1_ complex are highly conserved in almost all plant cells. Our study provides an intriguing example that the necrotrophic pathogen secretes a small protein which might attack the well conserved component of mitochondrial respiratory chain in plant cells. This hypothesis is also consistent with the broad host range of *S*. *sclerotiorum*.

In summary, we screened for small, secreted proteins that were significantly up-regulated during infection and identified a "toxin-like" and "effector-like" protein SsSSVP1 in *S*. *sclerotiorum*. *SsSSVP1* is essential for the full virulence of *S*. *sclerotiorum*. SsSSVP1^∆SP^ interacts with QCR8 and hijacks QCR8 into the cytoplasm in plant cells. The SsSSVP1^∆SP^-QCR8 interaction disturbs the location of QCR8 and hence might interfere with the biological functions of QCR8. The functional loss of QCR8 may seriously affect the plant energy metabolism and caused significant cell death. Two cysteine residues at 38 and 44 of SsSSVP1 are crucial for its structure and functions. These findings further enhance our understanding of the pathogenic mechanism of *S*. *sclerotiorum*, highlighting the necessity for large-scale screening and function analyses of the effector candidates in typical necrotrophic fungi with broad host ranges.

## Materials and Methods

### Bacterial and fungal strains, plants, culture conditions and transformation of *S*. *sclerotiorum* and *N*. *benthamiana*

The virulent *S*. *sclerotiorum* wild-type strain Ep-1PNA367 [63] and *B*. *cinerea* wild-type strain B05.10 were used in this study. Fungal cultures were grown on potato dextrose agar (PDA, Difco, Detroit, MI, USA) or inoculated in CM liquid medium at 20°C. *S*. *sclerotiorum* and *B*. *cinerea* transformants were cultured on PDA amended with 80 μg/ml hygromycin B (Calbiochem, San Diego, CA) to stabilize the transformants. *Escherichia coli* strain JM109 and DH5α was used to propagate all plasmids, and *A*. *tumefaciens* strains EHA105 and GV3101 were used for the transformation of fungi and plants, respectively. Seedlings from *A*. *thaliana* (ecotype Columbia-0) and *N*. *benthamiana* were grown in the greenhouse at 20 ± 2°C under a 12 h light/dark cycle with 70% relative humidity. The canola cultivar used for virulence assay was zhongyou 821 [64], which is slightly resistant to *S*. *sclerotiorum*. The *Agrobacterium*-mediated transformation method was used to transform *S*. *sclerotiorum* as previously described [65], with a modification related to *Agrobacterium* cultivation: the *A*. *tumefaciens* cells were not diluted in minimal medium and directly cultured in induction medium for co-cultivation. The *Agrobacterium*-mediated transformation method was performed to transform *N*. *benthamiana* via infiltration according to published protocols [66].

### Bioinformatics analysis

The publicly available genomic sequence database of *S*. *sclerotiorum* 1980 UF-70 (http://www.broadinstitute.org/annotation/genome/sclerotinia_sclerotiorum/MultiDownloads.html) was used to characterize all *S*. *sclerotiorum* genes examined in this study. SignalP was used to identify secreted proteins and their SPs [67]. BlastP analysis was done on the website of NCBI (http://www.ncbi.nlm.nih.gov/). The amino acid sequences were aligned using COBALT [68] and viewed and edited in Jalview [69]. The DGE analysis and the identification of differentially expressed genes were performed according to our previous study [35].

### Vector construction for gene expression and gene silencing in *S*. *sclerotiorum* and *N*. *benthamiana*

To generate SsSSVP1-FLAG fusion construct (S9A Fig), the promoter P_EF-1α_ was PCR amplified using the primers P_EF-1α_ F/R and subsequently digested with *Xho* I and *Sac* I. The PCR products of *SsSSVP1* were amplified with the primers SsSSVP1-FLAG F/R and subsequently digested with *Sac* I and *Sma* I. These two fragments were sequentially ligated into the pCH vector [65] through the formation of intermediate constructs. Based on our experience in gene silencing in *S*. *sclerotiorum*, the silencing efficiency of different RNAi strategies varies from gene to gene. To obtain knockdown transformants with a higher silencing efficiency, two RNAi strategies described by Nguyen [70] and Yu *et al*. [65] were adopted to construct the *S*. *sclerotiorum* RNAi vectors: a 320 bp fragment from *SsSSVP1* was amplified with the primers RNAi-SsSSVP1 F/R from the *S*. *sclerotiorum* cDNA library and (i) directly ligated into the digested pCXDPH vector at the *Xcm* I (New England Biolabs, Beverly, MA, USA) site to produce pRNAi-1 vector (S9B Fig) or (ii) digested with suitable enzymes and subsequently ligated into pCIT [27] between PtrpC, the intron and TtrpC in the opposite orientation via intermediate vectors. Subsequently, the PtrpC-intron-TtrpC fragment containing the two *S*. *sclerotiorum* gene fragments in the opposite orientation was digested with *Sac* I and *Xho* I and subsequently ligated into pCH to produce pRNAi-2 vector (S9C Fig). Both of these two different RNAi strategies were used to silence *SsSSVP1* and similar results were obtained. The transformants used in this study were produced using pRNAi-2 vector. All the constructs were confirmed through sequencing analysis. The primers are shown in S2 Table. These constructs were then introduced into the *A*. *tumefaciens* strain EHA105 through electroporation [71].

For *SsSSVP1* constitutive expression in *N*. *benthamiana*, the recombinant TRV-based *A*. *tumefaciens* binary virus vectors pTRV1 and pTRV2 [39] were used for gene expression and gene silencing in *N*. *benthamiana* in this study. To generate the constitutive expression constructs, (i) SsSSVP1 with and without the SP-encoding sequences were amplified using the primers pTRV-SsSSVP1 F/R and pTRV-SsSSVP1^∆SP^ F/R, respectively; (ii) GFP with and without the SP-encoding sequences were amplified using the primers pTRV-GFP F/R and pTRV-SP-GFP F/R, respectively; (iii) SsSSVP1^∆SP^ and GFP-encoding sequence were amplified using the primers SsSSVP1^∆SP^ F/R and GFP F/R, respectively. The PCR products were subsequently digested with the appropriate restriction enzymes, followed by ligation to the intermediate vector pBI121 [72], and then the PCR product of the SsSSVP1^∆SP^-GFP fusion was amplified from the recombinant pBI121 using the primers pTRV-SsSSVP1^∆SP^-GFP F/R; (iv) The PCR product of the SsSSVP1-GFP fusion was amplified using the primers pTRV-SsSSVP1-GFP F and GFP R from the pTRV2-SsSSVP1^∆SP^-GFP constructs. The final fragments from (i) to (iv) were directly cloned into pTRV2 digested with *Xcm* I to construct pTRV2-SsSSVP1^∆SP^, pTRV2-SsSSVP1, pTRV2-GFP, pTRV2-SP-GFP, pTRV2-SsSSVP1^∆SP^-GFP and pTRV2-SsSSVP1-GFP vectors, respectively (S9D–S9I Fig). For the co-localization assay, SsSSVP1^∆SP-C38A-C44A^-mCherry fusion protein encoding sequence was constructed by spliced overlap extension PCR. SsSSVP1^∆SP^-mCherry fusion protein encoding sequence was amplified using the primers pTRV-SsSSVP1^∆SP^-mCherry F/R from the construct containing SsSSVP1-mCherry-NLS. QCR8 and GFP encoding sequences were amplified using the primers QCR8-F/R and GFP F/R, respectively, and then cloned into the intermediate vector pBI121, from which the QCR8-GFP fusion protein encoding sequence was amplified using the primers pTRV-QCR8-GFP F/R. The amplified SsSSVP1^∆SP-C38A-C44A^-mCherry, SsSSVP1^∆SP^-mCherry and QCR8-GFP fusion protein encoding sequences were finally cloned into the pTRV2 vector, respectively, using the same method described as above (S9J and S9K Fig). For validating the expression of SsSSVP1^∆SP-C38A-C44A^ and SsSSVP1^∆SP^ in tobacco leaf cells using western blotting analysis, the primers pTRV2-SsSSVP1-3×FLAG F/R were used to amplify SsSSVP1^∆SP-C38A-C44A^ and SsSSVP1^∆SP^ from the constructs containing these two fragments, respectively, before they were cloned into the pTRV2 vector (S9L Fig). Constructs containing these fragments in the correct orientation were PCR screened using the primer pTRV F and corresponding downstream primers respectively. To generate the VIGS-pTRV2-QCR8 silencing constructs (S9M and S9N Fig), partical coding regions of the three *QCR8* genes were amplified from the cDNA liabrary of *N*. *benthamiana* using the primers RNAi-QCR8-1 F/R, RNAi-QCR8-2 F/R and RNAi-QCR8-3 F/R, respectively, and then digested with *EcoR* І and *BamH* І prior to be ligated into VIGS-pTRV2 vector digested with the same pair of restriction enzymes. The pTRV1 construct (S9O Fig) from Liu Y et al. [39] was directly used. All the constructs were confirmed through sequencing analysis. The primers are shown in S2 Table. These constructs were then introduced into the *A*. *tumefaciens* strain GV3101-pM90 through electroporation [71]. Equal amounts of agrobacterium containing the constructs and pTRV1 were mixed respectively for infiltration performed with *N*. *benthamiana* leaves as previously described [28].

### Protein precipitation, dialysis and condensation

To determine whether SsSSVP1 was secreted into the liquid cultures, the positive SsSSVP1-FLAG engineered strains were cultured in liquid CM medium at 20°C for 3 days, with shaking at 200 rpm. The culture broth was filtered with 4 layers of Calbiochem Miracloth and centrifuged at 10,000 rpm for 5 min to remove the hyphal fragments. Secreted proteins in the fermentation liquid were precipitated with solid ammonium sulfate (100% saturated). The precipitated secreted proteins were dissolved in PBS buffer (137 mM NaCl, 2.7 mM KCl, 10 mM Na_2_HPO_4_, and 2 mM KH_2_PO_4_) and desalinated through dialysis. The protein extracts in dialysis bag were further condensed using saccharose at 4°C and lyophilized overnight before being dissolved in 0.1ml of 4×protein loading buffer for western blot analysis after quantification.

### Western blotting

To screen the positive SsSSVP1-FLAG engineered strains, total proteins extracted from the mycelia of SsSSVP1-FLAG transformants by cell lysis buffer (Beyotime, Wuhan, Hubei, China) were used for immunoprecipitation (IP) and western blot analysis. About 5 μl ANTI-FLAG M2 monoclonal antibody (Sigma, Saint Louis, Missouri, USA) was added to 1 ml protein extracts and then was incubated at room temperature for 2 hours. Afterwards, protein A+G agarose (Beyotime, Wuhan, Hubei, China) was added to the protein extracts and was incubated at room temperature for 1 hour before it was collected by centrifugation and washed for five times by the cell lysis buffer, and then protein loading buffer was added for following western blot analysis. Proteins were separated by SDS-PAGE gel (12%) before they were transferred onto a 0.22 μm PVDF membrane (Millipore) using a Trans-Blot SD Semi-Dry Electrophoretic Transfer Cell (Bio-Rad). A monoclonal α-Anti FLAG M2 antibody (Sigma-Aldrich, St. Louis, MO, USA) and a goat anti-mouse IgG conjugated with alkaline phosphatase (Sigma-Aldrich, St. Louis, MO, USA) were used as a primary antibody and a secondary antibody respectively. To validate the secretion of SsSSVP1, total proteins obtained from the liquid CM medium through protein precipitation, dialysis and condensation described as above were directly used for western blot analysis using the same method without the IP procedure. The secondary antibody used in this experiment was a goat anti-mouse IgG conjugated with horseradish peroxidase (HRP) (Sigma-Aldrich, St. Louis, MO, USA). The signals of blots were detected using Pierce ECL Western Blotting Substrate (Thermo Scientific).

### Nuclear targeting assay

To generate the *SsSSVP1*-mCherry-NLS and SP-mCherry-NLS fusion constructs, *SsSSVP1* and mCherry were PCR amplified using the primers SsSSVP1 F/R and mCherry F/R respectively. The PCR products were digested with appropriate restriction enzymes and then ligated into the pCXH (a fungal expression vector constructed by our lab) through the formation of intermediate constructs. The SP-mCherry-NLS and SsSSVP1-mCherry-NLS fragments with a stop codon were amplified from the pCXH vector with *SsSSVP1*-mCherry fusion using the primers SP-mCherry-NLS F/R and SsSSVP1-mCherry-NLS F/R before they were finally cloned into pDL2 vector [73], respectively, by the yeast gap repair approach [74]. The SP-mCherry-NLS and *SsSSVP1*-mCherry-NLS fusion constructs were transformed into the *B*. *cinerea* B05.10 strain using the PEG-mediated transformation method [75]. Tissues from onion bulb lower epidermal cells infected with the *B*. *cinerea* engineered strains expressing SP-mCherry-NLS and SsSSVP1-mCherry-NLS fusion proteins were examined at 48 hpi, respectively.

### Confocal microscopy

To observe fluorescence, the tobacco tissues were harvested from infiltrated tobacco leaves at 3 dpi and the onion tissues were harvested from inoculated lower epidermis at 36 hpi, and then directly imaged under a confocal laser scanning microscope (OLYMPUS microscope FV1000). The 488-, 587, 514- and 458-nm absorption laser lines with corresponding appropriate specific emission filter sets were used when images of GFP, mCherry, YFP and chloroplast autofluorescence were recorded, respectively.

### Nucleic acid isolation and transcript level determination

Genomic DNA was isolated as previously described [76] and used for the validation of T-DNA insertion in the transformants through PCR with the primers Hyg F/R (S2 Table). To evaluate the expression levels of *SsSSVP1* in different transformants, the transformants were inoculated on cellophane placed on PDA plates before pure mycelia of the transformants were collected for RNA isolation. To evaluate the expression levels of *SsSSVP1* during different infection stages of the wild-type strain, pure fresh mycelia without culture medium were inoculated on *A*. *thaliana* leaves. The inoculated leaves were collected at 0, 3, 6, 9, 12 hpi and frozen in liquid nitrogen and ground to a powder for RNA extraction. To evaluate the expression levels of *QCR8*, the upper and middle *N*. *benthamiana* leaves were sampled one month after *A*. *tumefaciens* infiltration. Total RNA was extracted using the TRIZOL Reagent (Huashun Bioengineering Co, Shanghai, China) according to the manufacturer’s instructions and treated with DNase I (RNase free, Takara, Dalian, China). Synthesis of first-strand cDNA and qRT-PCR were conducted according to Zhu *et al*. [27]. The expression levels of *SsSSVP1* were examined through qRT-PCR using the primers QPCR-SsSSVP1 F/R. The expression levels of the *S*. *sclerotiorum* β-tubulin gene (SS1G_04652) [77] were used to normalize the expression of *SsSSVP1* in each corresponding qRT-PCR sample using the primers Tub F/R. The expression levels of the three genes encoding the homologs of QCR8 in *N*. *benthamiana* were examined through qRT-PCR using the primers QPCR-QCR8-1 F/R, QPCR-QCR8-2 F/R and QPCR-QCR8-3 F/R, respectively. The expression levels of the *N*. *benthamiana* actin gene (AY179605.1) [27] were used to normalize the expression of *QCR8* in each corresponding qRT-PCR sample using the primers Actin F/R. The qRT-PCR assay was repeated at least twice for each gene, with three replicates. The primers used for qRT-PCR are shown in S2 Table.

### Characterization of the *SsSSVP1*-silenced and *SsSSVP1*-overexpressed transformants

The detached *B*. *napus* (zhongyou 821) leaves under the same physiological conditions were used for the virulence assay of *S*. *sclerotiorum* wild-type strain and transformants. To evaluate virulence, at least six individual detached *B*. *napus* leaves or *in vivo A*. *thaliana* leaves were inoculated with a single 0.5-cm diameter mycelium-colonized agar plug obtained from the expanding margins of PDA-cultured colonies. Inoculated leaves were maintained at 100% relative humidity at 20°C for 48 h (for *B*. *napus* leaves) or 36 h (for *A*. *thaliana* leaves). Disease severity was measured using the average lesion diameter. To assay growth rates, the wild-type strain and the transformants were cultivated on PDA at 20°C for 3 days. Mycelial agar discs were collected from the active colony edge and inoculated in the center of the PDA Petri dish at 20°C before the hyphal growth was examined. Each experiment was performed independently at least three times.

### Protein-protein interaction assays

Y2H analysis was performed using a GAL4-based Y2H system (Matchmaker Gold Systems; Clontech, Palo Alto, CA). The construction of Y2H library, autoactivation and toxicity test and the screening of Y2H library were performed according to the manufacturer’s instructions. The primers used to create the corresponding constructs are listed in S2 Table. The bait and prey plasmids were co-transformed into a yeast strain Y2HGold (Clontech, Palo Alto, CA). Yeast transformation was performed according to the manufacturer’s instructions. The transformants were assayed for growth on synthetic dropout (SD)/-Trp-Leu plates, and cultured on liquid synthetic SD/-Trp-Leu medium for 36 hours before being collected by centrifugation. The concentration of collected yeast cells were adjusted to 10^6^ (cells/ml) using sterile water, and then 5 μl yeast suspension was assayed for growth on SD/-Trp-Leu-His-Ade plates containing the X-α-gal and Aureobasidin A (AbA).

For Co-IP assay, to construct pCNF3-SsSSVP1^∆SP^-GFP and pCNF3-QCR8-3×FLAG (S9P and S9Q Fig), the full-length of the SsSSVP1^∆SP^-GFP and QCR8-3×FLAG were amplified using the specific primers COIP-SsSSVP1^∆SP^-GFP F/R and COIP-QCR8-3×FLAG F/R (S2 Table), respectively, and then cloned into the pCNF3 vector (a plant expression vector constructed by our lab). *A*. *tumefaciens* containing the pCNF3-SsSSVP1^∆SP^-GFP and pCNF3-QCR8-3×FLAG constructs were co-infiltrated into *N*. *benthamiana* leaves using the same method described as above. Total protein was isolated by homogenizing tissues with RIPA lysis buffer (Beyotime, Wuhan, Hubei, China) with a modification [50 mM Tris pH7.4, 150 mM NaCl, 1% NP-40, 0.25% sodium deoxycholate, 1 mM sodium orthovanadate, 1 mM sodium fluoride, 1 mM EDTA, 0.5 μg/ml leupeptin, 1 mM phenylmethanesulfonyl fluoride (PMSF) and 1% proteinase inhibitor cocktail (Sigma, Saint Louis, Missouri, USA)]. Approximately 3 g plant tissues were lysed by 10 ml RIPA lysis buffer. The total protein was then centrifuged at 13000 rpm for 1 h to remove residues. For anti-GFP IP, approximately 2 ml supernatant RIPA lysis buffer containing the total protein was incubated with 10 μl of anti-GFP monoclonal antibody (sigma, Saint Louis, Missouri, USA) and 50 μl of protein G plus-Agarose (Santa Cruz Biotechnology, Inc. Dallas, Texas, USA) for 8 h at 4°C on a rotary shaker. The beads were then collected and washed five times with RIPA lysis buffer. The bound protein was eluted from beads by boiling in protein sample buffer. One third of the immunoprecipitated protein was subjected to immunoblot analyses with anti-FALG monoclonal antibody (sigma, Saint Louis, Missouri, USA). Approximately 25 μl of RIPA lysis buffer containing the total protein was loaded as input control.

BiFC assay was used to study the interaction of SsSSVP1^∆SP^ and QCR8 based on a previously described method [78]. To construct the pBISPYNE-SsSSVP1^∆SP^ and pBISPYCE-QCR8 vectors (S9R and S9S Fig), respectively, the full-length cDNAs of the SsSSVP1^∆SP^ and QCR8 were amplified using the specific primers BiFC-SsSSVP1^∆SP^ F/R and BiFC-QCR8 F/R (S2 Table), respectively, recombined with the N- and C-termini of YFP, respectively, and subsequently cloned into the pBI121 vector through intermediate vectors pUC-SPYNE and pUC-SPYCE. The constructs were verified by sequencing. All plasmids were transformed into *N*. *benthamiana* leaves via the *A*. *tumefaciens* strain GV3101-pM90.

### Site-directed mutagenesis

The eight cysteine residues of SsSSVP1^∆SP^ were substituted by alanine respectively according to the manual of QuikChange II XL Site-Directed Mutagenesis Kit (Stratagene). The double-point mutant SsSSVP1^∆SP-C38A-C44A^ was constructed by fusion PCR using the primers Mut^C38A-C44A^-1 F/R and Mut^C38A-C44A^-2 F/R. The coding sequences of SsSSVP1^∆SP^ mutants were cloned into the pGBKT7 and pGADT7 vector respectively for Y2H analysis and cloned into the pTRV2 vector respectively for functional analysis. Mutations were confirmed by sequencing analysis. The primers used in this experiment were listed in S2 Table.

## Supporting Information

S1 Fig The occasional subcellular localization of SsSSVP1^∆SP^ in plant cells and the co-localization of SsSSVP1^∆SP^ and QCR8. (**A**) Laser confocal micrograph showing SsSSVP1^∆SP^ occasionally localizes in nuclei and cytoplasmic compartments in a particle-like form. These photos were taken from different areas in the same *N*. *benthamiana* leaf. Red particles showed chloroplast autofluorescence. Photos were taken 3 days after agroinfiltration. Maximum projections of 4 confocal images captured along the z-axis are shown. (**B**) SsSSVP1^∆SP^ and QCR8 occasionally co-localize in nuclei or cytoplasm in a particle-like form. These photos were taken from different areas in the same *N*. *benthamiana* leaf. Fluorescence was monitored 3 days after agroinfiltration using confocal laser scanning microscopy. The images show maximum projections of 4 confocal images captured along the z-axis.
(TIFF)

S2 Fig The ER-like fluorescence distribution of SP-GFP and SsSSVP1-GFP. Both SP-GFP (which was used as control) and SsSSVP1-GFP localized in ER-like structure in plant cells. The left column fluorescence images, which are higher magnification images of the areas marked by the red boxes in the right column, indicated ER-like structure. The SP refers in particular to the SP of SsSSVP1. Photos were taken 3 days after agroinfiltration.
(TIFF)

S3 Fig The fluorescence distribution of SsSSVP1-mCherry-NLS in different layers of host cells. Details for this nuclear targeting assay see Fig 4. (**A**) The diagram of laser layer-by-layer scanning around z-axis by a confocal microscope. (**B**) Divided layer images of laser scanning. All the divided layer images were merged finally. Different layers of the intact surrounding cells were checked independently to ensure there were no hyphae in these cells. Areas within yellow dotted line indicate hyphal invaded onion epidermal cells.
(TIFF)

S4 Fig *SsSSVP1*-silenced transformants showing reduced virulence on *A*. *thaliana* leaves. (**A**) Virulence test of *SsSSVP1*-silenced transformants on *in vivo A*. *thaliana* leaves. (**B**) Virulence was evaluated according to the lesion diameter at 36 hpi. Six independent replicates were performed. The values are presented as the means±s.d. Different letters on the graph indicate statistical differences, P = 0.05.
(TIFF)

S5 Fig The biological characteristics of the *SsSSVP1* over-expression transformants *SsSSVP1*. (**A**) The colony morphology of the *SsSSVP1* over-expression transformants. Colonies were grown on PDA for 10 days at 20°C. (**B**) No significant virulence reduction is observed in over-expression transformants of *SsSSVP1*. Virulence is evaluated on detached oilseed rape leaves (*B*. *napus* zhongyou 821) according to the lesion diameter at 20°C for 48 h. (**C**) The relative expression of *SsSSVP1* in different over-expression transformants is analyzed through qRT-PCR. β-tubulin expression levels is used to normalize the expression levels of *SsSSVP1* in different samples, and the expression level in the wild-type strain was set as 1.0. (**D**) Western blot analysis with proteins isolated from mycelia of the wild-type strain and the SsSSVP1-FLAG engineered strains respectively. SDS-polyacrylamide gel electrophoresis shows the equal loading amount of proteins used for the west blot analysis. Alkaline phosphatase conjugated secondary antibody detected an approximate 17 kDa band in OESsSSVP1-3, but not in Ep-PNA367. (**E**) Comparison of the lesion diameter of over-expression transformants and the wild-type strain. (**F**) Comparison of the growth rate of over-expression transformants and the wild-type strain. In all experiments, three independent replicates were performed. The values are presented as the means±s.d. Different letters on the graph indicate statistical differences, P = 0.05.
(TIFF)

S6 Fig Y2H assay showed SsSSVP1^∆SP^ interacted with the other homolog of QCR8 (AT-QCR8, AT5G05370) in *A*. *thaliana* and all the homologs of QCR8 (NB-QCR8) in *N*. *benthamiana*. pGBKT7-53 and pGADT7-T were used as positive controls (Clontech). “-” means there is an empty vector. The negative controls indicated SsSSVP1^∆SP^ and QCR8 were not self-activated. Photos were taken 2 dpi.
(TIFF)

S7 Fig Y2H assay and single site-directed mutagenesis of the eight cysteines in SsSSVP1^∆SP^ were combined to screen double-point mutants losing the capability to form homo-dimer. The coding sequences of different single-point mutants of SsSSVP1^∆SP^ were cloned into pGBKT7 and pGADT7 vector, respectively, before performing Y2H assay. Bottom left of the slash shows the growth of co-transformed Y2H strain on SD/-Leu-Trp medium and top right of the slash shows the growth of co-transformed Y2H strain on SD/-Leu-Trp-His-Ade+X-α-Gal+AbA medium. Red rectangle indicates SsSSVP1^∆SP-C38A^ cannot interact with SsSSVP1^∆SP-C44A^ anymore. Photos were taken 2 dpi.
(TIFF)

S8 Fig The validation of the effectiveness of the TRV-VIGS system. The phenotype of *PDS*-silenced *N*. *benthamiana* lines using TRV based VIGS system. Silencing of the *PDS* in *N*. *benthamiana* plants caused photobleaching phenotype. No obvious phenotype was observed in the control. Photos were taken one month after *A*. *tumefaciens* infiltration.
(TIFF)

S9 Fig Graphical representations of the vectors and constructs used in this study. (**A**) Graphical representation of SsSSVP1-FLAG fusion construct used for immunolocalization. The SsSSVP1-FLAG fusion was expressed under the control of the P_EF-1α_ promoter and the T*trpC* terminator. (**B**) Construction of pRNAi-1 vector targeted against *SsSSVP1*. The *SsSSVP1* fragment was amplified using the corresponding primers from the *S*. *sclerotiorum* cDNA library and subsequently inserted between the *Neurospora crassa trpC* promoter P*trpC* and the *Aspergillus nidulans gpd* promoter P*gpd*. The P*trpC* and P*gpd* are in an opposite directions in this vector. (**C**) Construction of pRNAi-2 vectors targeted against *SsSSVP1*. The fused *SsSSVP1*-intron-*SsSSVP1* fragment was inserted between the *A*. *nidulans trpC* promoter P*trpC* and terminator T*trpC*. The two fragments of *SsSSVP1* are same but in a reverse orientation in this vector. The intron is from *Gibberella zeae*. (**D-L**) Construction of binary virus vectors pTRV2-SsSSVP1^∆SP^, pTRV2-SsSSVP1, pTRV2-GFP, pTRV2-SP-GFP, pTRV2-SsSSVP1^∆SP^-GFP, pTRV2-SsSSVP1-GFP, pTRV2-SsSSVP1^∆SP-C38A-C44A^-mCherry, pTRV2-SsSSVP1^∆SP^-mCherry, pTRV2-QCR8-GFP, pTRV2-SsSSVP1^∆SP-C38A-C44A^-3×FLAG and pTRV2-SsSSVP1^∆SP^-3×FLAG. Corresponding fragments were cloned and inserted into the TA cloning site in the pTRV2 vector under the control of CSp, which is the promoter of the TRV coat protein. The open reading frame (ORF) originating from the virus correspond to a coat protein (CP). (**M** and **N**) Graphical representation of the VIGS-pTRV2 vectors used for silencing plant genes. The CSp promoter was removed from the pTRV2 vector to produce VIGS-pTRV2 vector. Partial fragments of *PDS* and *QCR8* amplified from the *N*. *benthamiana* cDNA library were cloned into the VIGS-pTRV2 vector, respectively, to silence corresponding endogenous genes in *N*. *benthamiana* plants. (**O**) Graphical representation of pTRV1 vectors. The open reading frames (ORFs) of pTRV1 originating from the virus correspond to 134 and 194 kDa replicases (RPs), a movement protein (MP) and a 16-kDa cysteine-rich protein, respectively. All TRV cDNA clones were placed between the duplicated CaMV 35S promoter (2×35S) and the nopaline synthase terminator (NOSt) in a T-DNA vector. Rz, self-cleaving ribozyme. (**P** and **Q**) Graphical representation of the pCNF3-SsSSVP1^∆SP^-GFP and pCNF3-QCR8-3×FLAG vectors used for Co-IP. (**R** and **S**) Graphical representation of the pBISPYNE-SsSSVP1^∆SP^ and pBISPYCE-QCR8 vectors used for BiFC. The SsSSVP1^∆SP^-YFP N-terminal fusion and the QCR8-YFP C-terminal fusion were expressed respectively under the control of the CaMV 35S promoter and the NOS terminator. SP indicates the SP of SsSSVP1. The Gly linker encodes three glycines in tandem. LB and RB refer to the left and right borders of the T-DNA, respectively.
(TIFF)

S1 Table The potential effector candidates in *S*. *sclerotiorum* identified in this study. **∆** The 314 potential effector candidates are designated as predicted secreted proteins, the gene expression of which is significantly up-regulated during infection of *S*. *sclerotiorum*. * indicates different types of predicted secreted proteins, please refer to Fungal Secretome Database (FSD) for details [79]. ** indicates the gene expression change folds detected by DGE.
(XLSX)

S2 Table Primers used in this study. (XLS)
