# Supplementary figures and images for: A Small Secreted Virulence-Related Protein Is Essential for the Necrotrophic Interactions of Sclerotinia sclerotiorum with Its Host Plants
Source: PLoS Pathog. 2016 Feb 1;12(2):e1005435. doi: 10.1371/journal.ppat.1005435 (PMC4735494; doi:10.1371/journal.ppat.1005435)

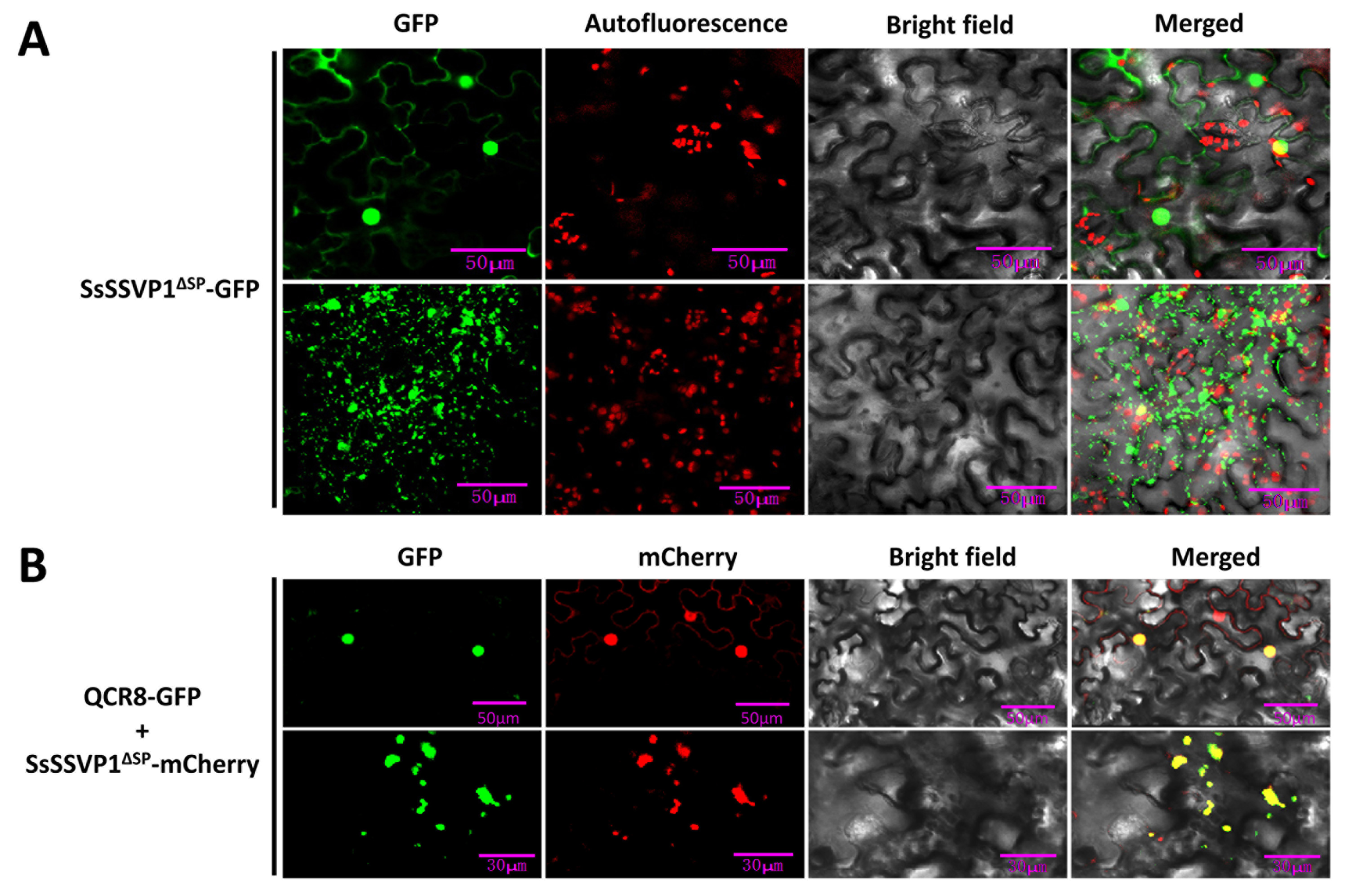

Supplement: S1 Fig — (A) Laser confocal micrograph showing SsSSVP1∆SP occasionally localizes in nuclei and cytoplasmic compartments in a particle-like form. These photos were taken from different areas in the same N. benthamiana leaf. Red particles showed chloroplast autofluorescence. Photos were taken 3 days after agroinfiltration. Maximum projections of 4 confocal images captured along the z-axis are shown. (B) SsSSVP1∆SP and QCR8 occasionally co-localize in nuclei or cytoplasm in a particle-like form. These photos were taken from different areas in the same N. benthamiana leaf. Fluorescence was monitored 3 days after agroinfiltration using confocal laser scanning microscopy. The images show maximum projections of 4 confocal images captured along the z-axis. (TIFF) [file ppat.1005435.s001.tiff]

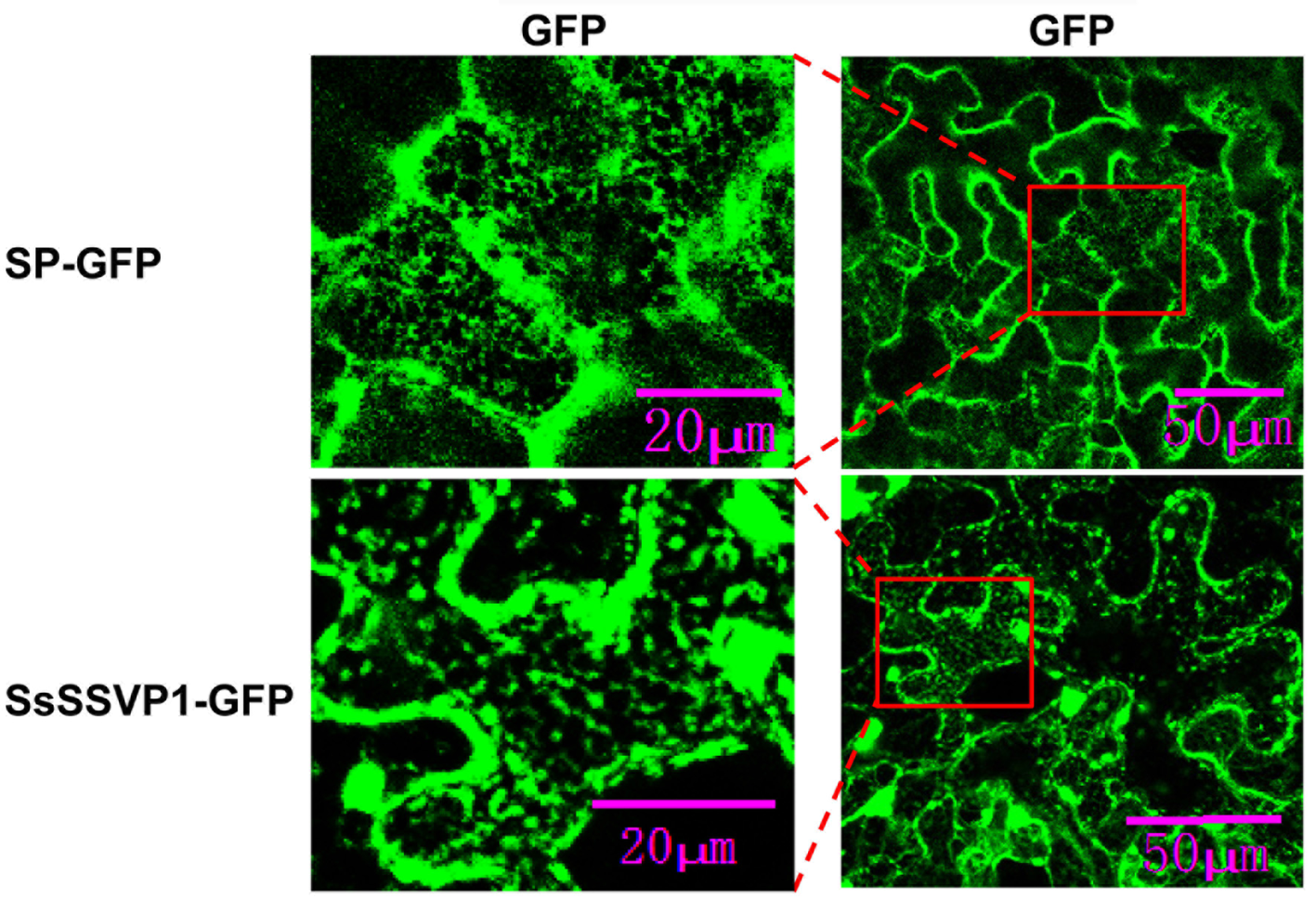

Supplement: S2 Fig — Both SP-GFP (which was used as control) and SsSSVP1-GFP localized in ER-like structure in plant cells. The left column fluorescence images, which are higher magnification images of the areas marked by the red boxes in the right column, indicated ER-like structure. The SP refers in particular to the SP of SsSSVP1. Photos were taken 3 days after agroinfiltration. (TIFF) [file ppat.1005435.s002.tiff]

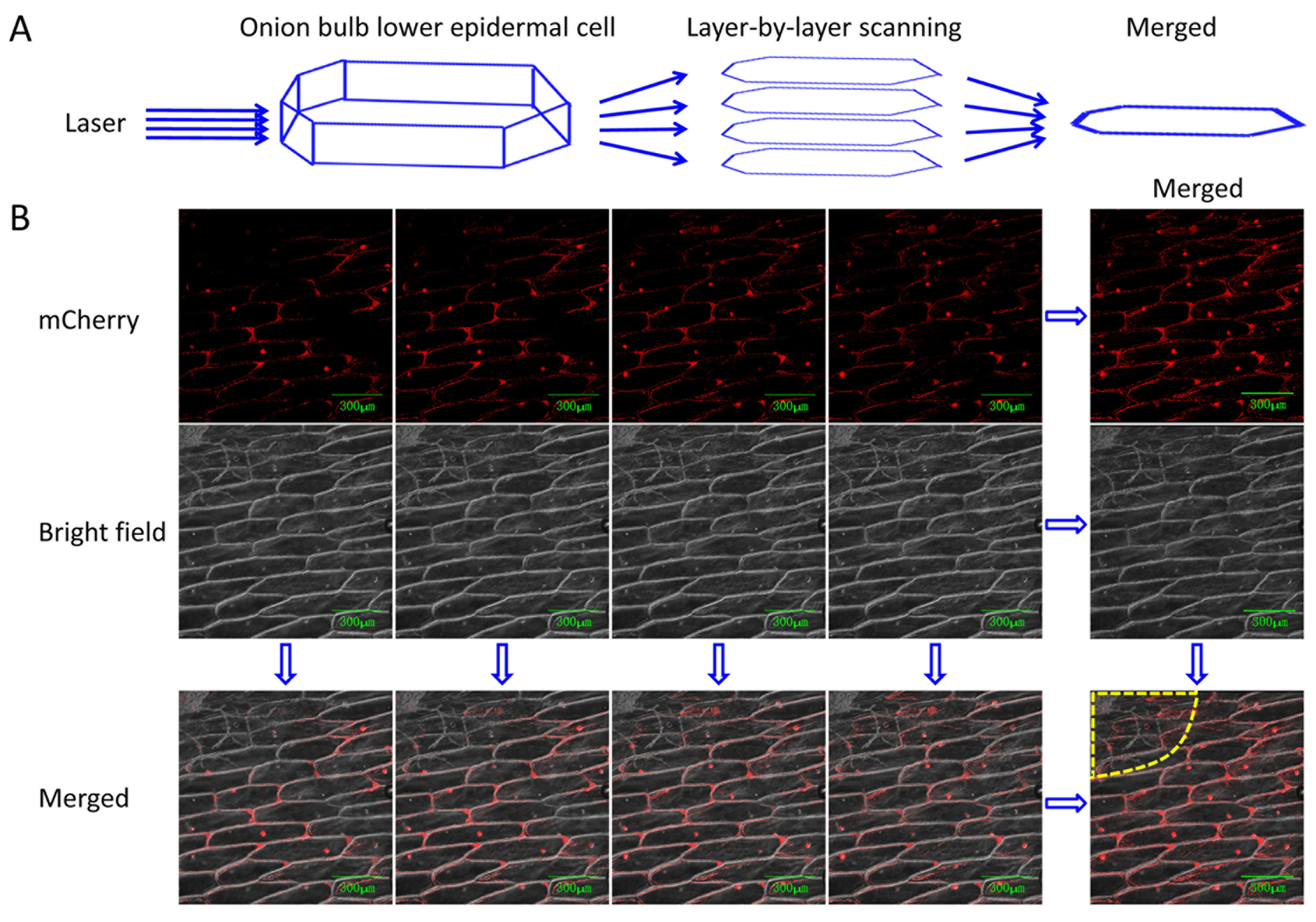

Supplement: S3 Fig — Details for this nuclear targeting assay see Fig 4. (A) The diagram of laser layer-by-layer scanning around z-axis by a confocal microscope. (B) Divided layer images of laser scanning. All the divided layer images were merged finally. Different layers of the intact surrounding cells were checked independently to ensure there were no hyphae in these cells. Areas within yellow dotted line indicate hyphal invaded onion epidermal cells. (TIFF) [file ppat.1005435.s003.tiff]

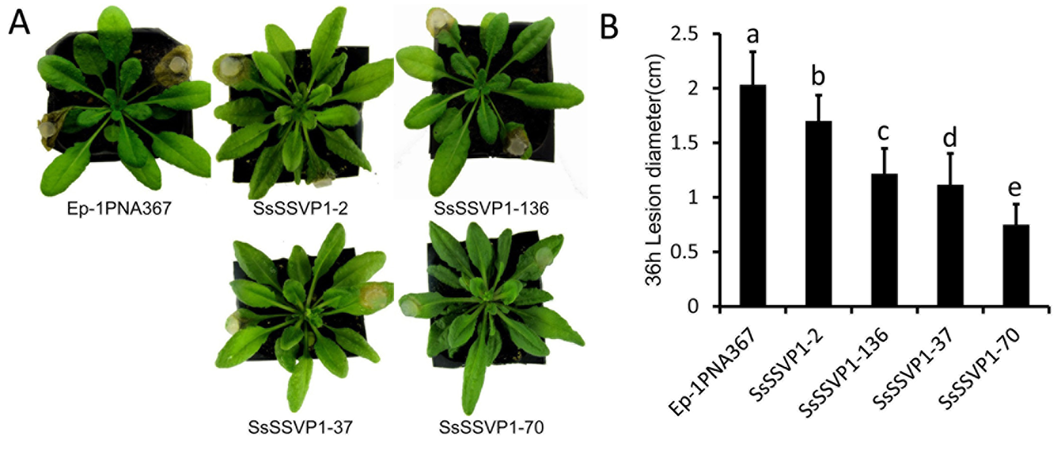

Supplement: S4 Fig — (A) Virulence test of SsSSVP1-silenced transformants on in vivo A. thaliana leaves. (B) Virulence was evaluated according to the lesion diameter at 36 hpi. Six independent replicates were performed. The values are presented as the means±s.d. Different letters on the graph indicate statistical differences, P = 0.05. (TIFF) [file ppat.1005435.s004.tiff]

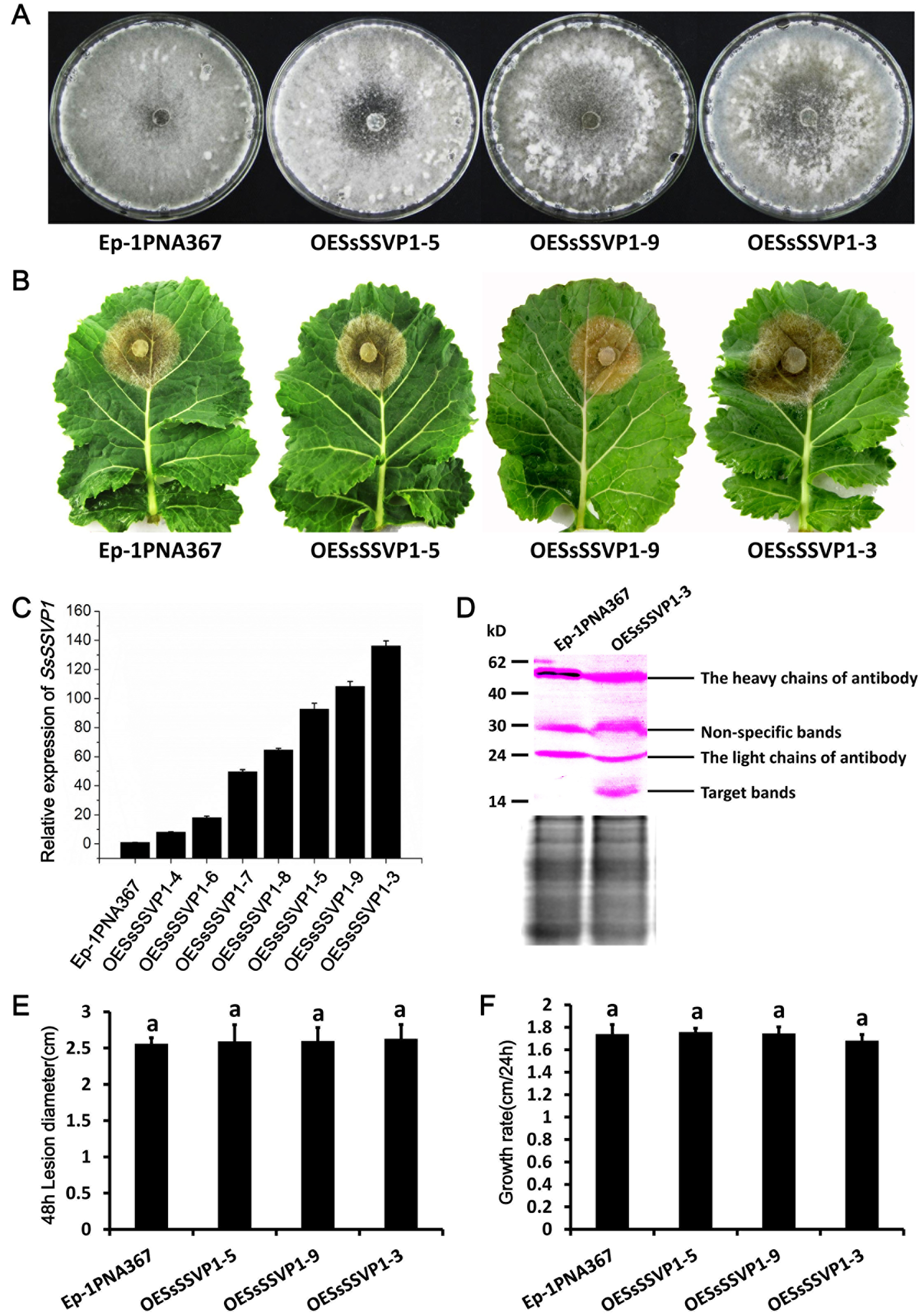

Supplement: S5 Fig — (A) The colony morphology of the SsSSVP1 over-expression transformants. Colonies were grown on PDA for 10 days at 20°C. (B) No significant virulence reduction is observed in over-expression transformants of SsSSVP1. Virulence is evaluated on detached oilseed rape leaves (B. napus zhongyou 821) according to the lesion diameter at 20°C for 48 h. (C) The relative expression of SsSSVP1 in different over-expression transformants is analyzed through qRT-PCR. β-tubulin expression levels is used to normalize the expression levels of SsSSVP1 in different samples, and the expression level in the wild-type strain was set as 1.0. (D) Western blot analysis with proteins isolated from mycelia of the wild-type strain and the SsSSVP1-FLAG engineered strains respectively. SDS-polyacrylamide gel electrophoresis shows the equal loading amount of proteins used for the west blot analysis. Alkaline phosphatase conjugated secondary antibody detected an approximate 17 kDa band in OESsSSVP1-3, but not in Ep-PNA367. (E) Comparison of the lesion diameter of over-expression transformants and the wild-type strain. (F) Comparison of the growth rate of over-expression transformants and the wild-type strain. In all experiments, three independent replicates were performed. The values are presented as the means±s.d. Different letters on the graph indicate statistical differences, P = 0.05. (TIFF) [file ppat.1005435.s005.tiff]

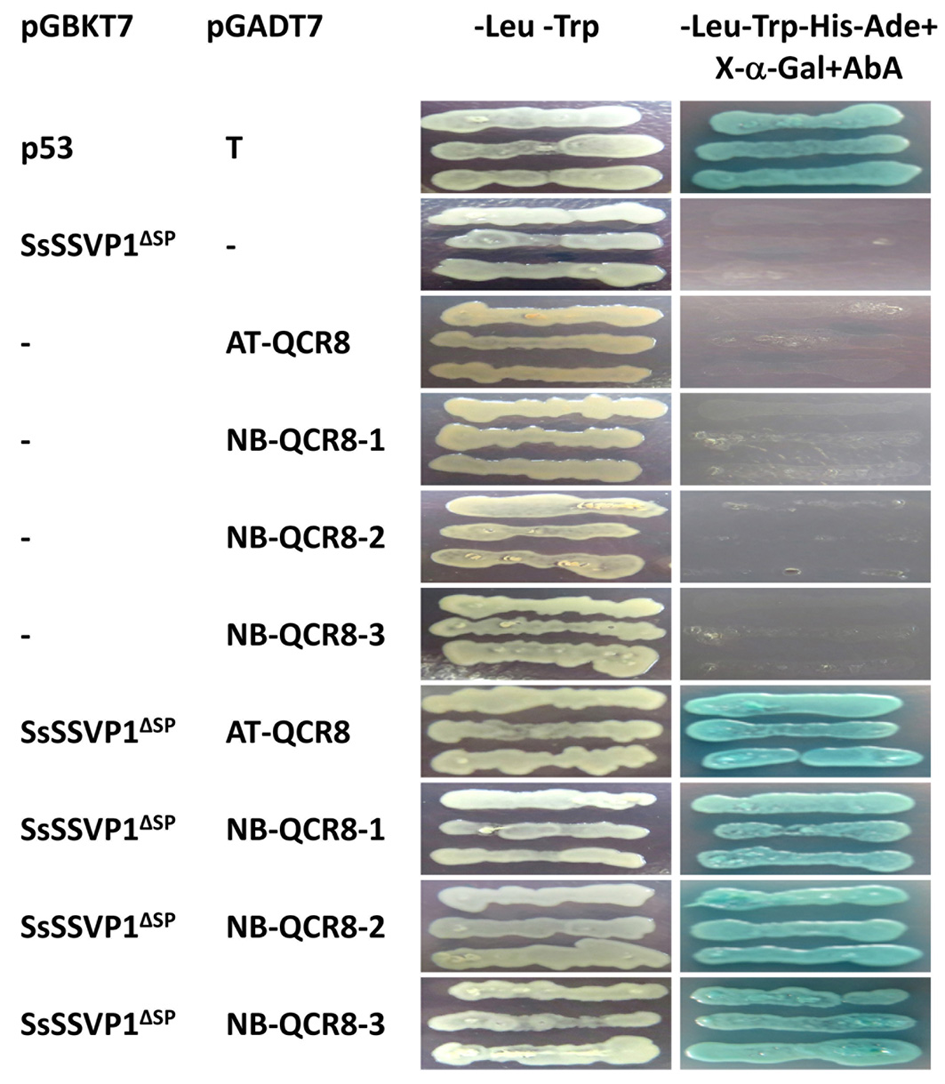

Supplement: S6 Fig — pGBKT7-53 and pGADT7-T were used as positive controls (Clontech). “-” means there is an empty vector. The negative controls indicated SsSSVP1∆SP and QCR8 were not self-activated. Photos were taken 2 dpi. (TIFF) [file ppat.1005435.s006.tiff]

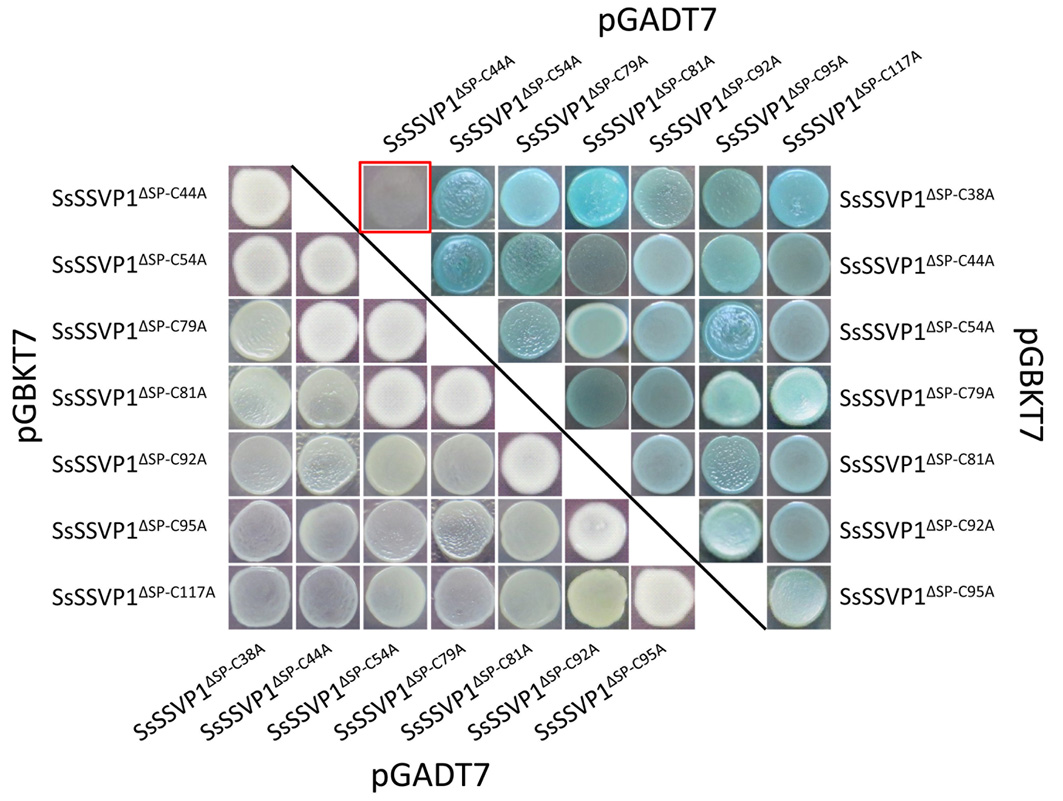

Supplement: S7 Fig — The coding sequences of different single-point mutants of SsSSVP1∆SP were cloned into pGBKT7 and pGADT7 vector, respectively, before performing Y2H assay. Bottom left of the slash shows the growth of co-transformed Y2H strain on SD/-Leu-Trp medium and top right of the slash shows the growth of co-transformed Y2H strain on SD/-Leu-Trp-His-Ade+X-α-Gal+AbA medium. Red rectangle indicates SsSSVP1∆SP-C38A cannot interact with SsSSVP1∆SP-C44A anymore. Photos were taken 2 dpi. (TIFF) [file ppat.1005435.s007.tiff]

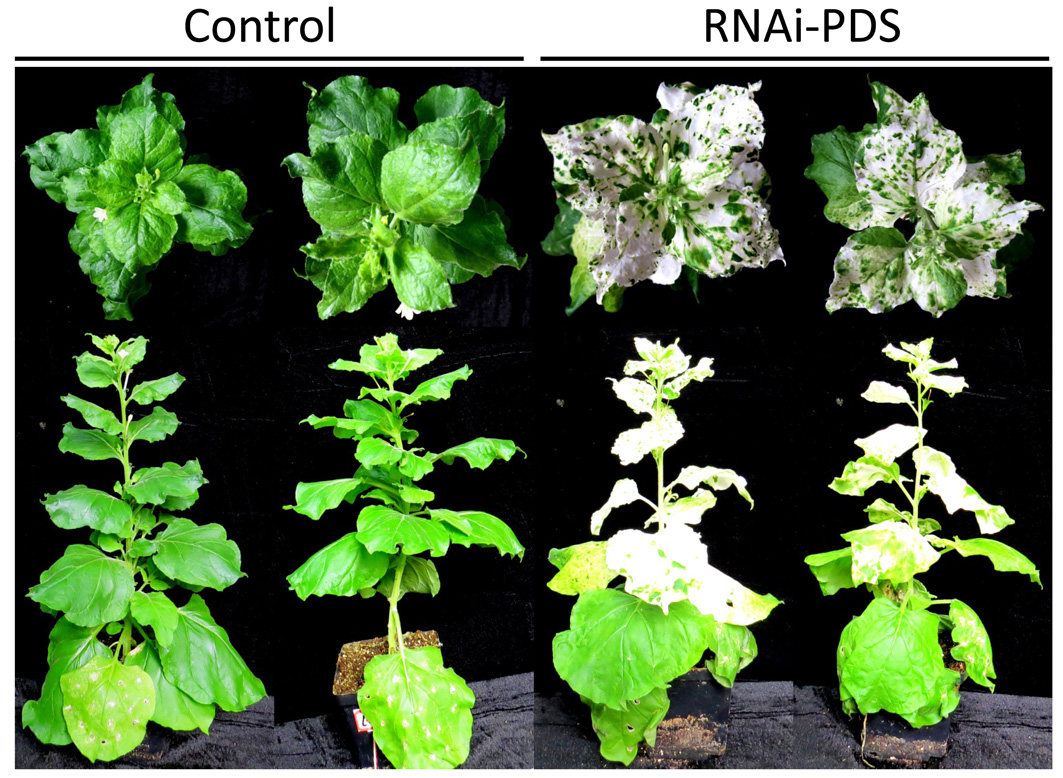

Supplement: S8 Fig — The phenotype of PDS-silenced N. benthamiana lines using TRV based VIGS system. Silencing of the PDS in N. benthamiana plants caused photobleaching phenotype. No obvious phenotype was observed in the control. Photos were taken one month after A. tumefaciens infiltration. (TIFF) [file ppat.1005435.s008.tiff]

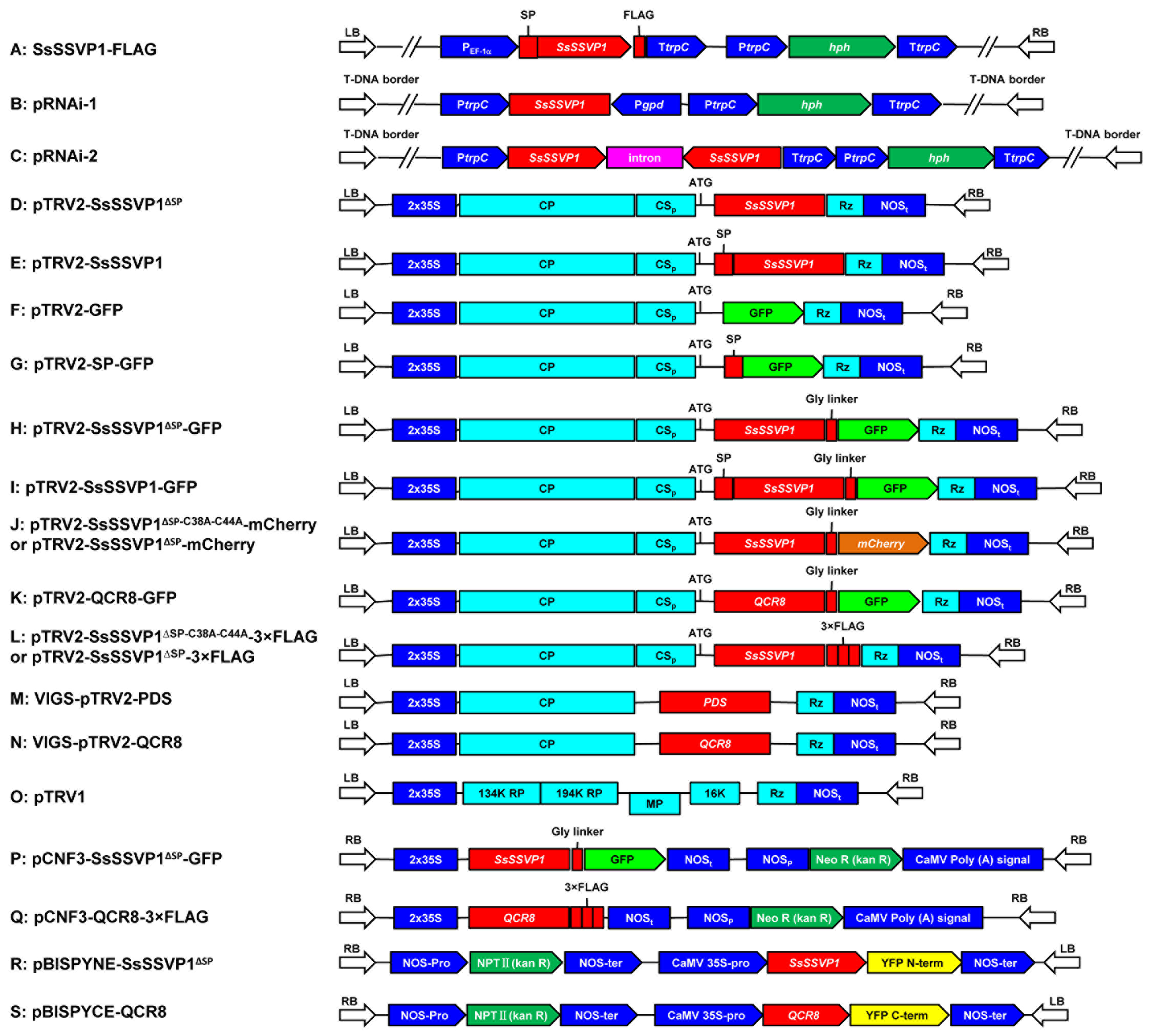

Supplement: S9 Fig — (A) Graphical representation of SsSSVP1-FLAG fusion construct used for immunolocalization. The SsSSVP1-FLAG fusion was expressed under the control of the PEF-1α promoter and the TtrpC terminator. (B) Construction of pRNAi-1 vector targeted against SsSSVP1. The SsSSVP1 fragment was amplified using the corresponding primers from the S. sclerotiorum cDNA library and subsequently inserted between the Neurospora crassa trpC promoter PtrpC and the Aspergillus nidulans gpd promoter Pgpd. The PtrpC and Pgpd are in an opposite directions in this vector. (C) Construction of pRNAi-2 vectors targeted against SsSSVP1. The fused SsSSVP1-intron-SsSSVP1 fragment was inserted between the A. nidulans trpC promoter PtrpC and terminator TtrpC. The two fragments of SsSSVP1 are same but in a reverse orientation in this vector. The intron is from Gibberella zeae. (D-L) Construction of binary virus vectors pTRV2-SsSSVP1∆SP, pTRV2-SsSSVP1, pTRV2-GFP, pTRV2-SP-GFP, pTRV2-SsSSVP1∆SP-GFP, pTRV2-SsSSVP1-GFP, pTRV2-SsSSVP1∆SP-C38A-C44A-mCherry, pTRV2-SsSSVP1∆SP-mCherry, pTRV2-QCR8-GFP, pTRV2-SsSSVP1∆SP-C38A-C44A-3×FLAG and pTRV2-SsSSVP1∆SP-3×FLAG. Corresponding fragments were cloned and inserted into the TA cloning site in the pTRV2 vector under the control of CSp, which is the promoter of the TRV coat protein. The open reading frame (ORF) originating from the virus correspond to a coat protein (CP). (M and N) Graphical representation of the VIGS-pTRV2 vectors used for silencing plant genes. The CSp promoter was removed from the pTRV2 vector to produce VIGS-pTRV2 vector. Partial fragments of PDS and QCR8 amplified from the N. benthamiana cDNA library were cloned into the VIGS-pTRV2 vector, respectively, to silence corresponding endogenous genes in N. benthamiana plants. (O) Graphical representation of pTRV1 vectors. The open reading frames (ORFs) of pTRV1 originating from the virus correspond to 134 and 194 kDa replicases (RPs), a movement protein (MP) and a 16-kDa cysteine-rich protein, respect [file ppat.1005435.s009.tiff]
